# PAK4 suppresses motor neuron degeneration in hSOD1^G93A^‐linked amyotrophic lateral sclerosis cell and rat models

**DOI:** 10.1111/cpr.13003

**Published:** 2021-02-21

**Authors:** Chaohua Cong, Weiwei Liang, Chunting Zhang, Ying Wang, Yueqing Yang, Xudong Wang, Shuyu Wang, Di Huo, Hongyong Wang, Di Wang, Honglin Feng

**Affiliations:** ^1^ Department of Neurology The First Clinical College of Harbin Medical University Harbin China; ^2^ Department of Neurology The Second Clinical College of Harbin Medical University Harbin China

## Abstract

**Objectives:**

Amyotrophic lateral sclerosis (ALS) is a fatal neurodegenerative disease characterized by the progressive loss of motor neurons (MN). CREB pathway‐mediated inhibition of apoptosis contributes to neuron protection, and PAK4 activates CREB signalling in diverse cell types. This study aimed to investigate PAK4’s effect and mechanism of action in ALS.

**Methods:**

We analysed RNA levels by qRT‐PCR, protein levels by immunofluorescence and Western blotting, and apoptosis by flow cytometry and TUNEL staining. Cell transfection was performed for in vitro experiment. Mice were injected intraspinally to evaluate PAK4 function in vivo experiment. Rotarod test was performed to measure motor function.

**Results:**

The expression and activation of PAK4 significantly decreased in the cell and mouse models of ALS as the disease progressed, which was caused by the negative regulation of miR‐9‐5p. Silencing of PAK4 increased the apoptosis of MN by inhibiting CREB‐mediated neuroprotection, whereas overexpression of PAK4 protected MN from hSOD1^G93A^‐induced degeneration by activating CREB signalling. The neuroprotective effect of PAK4 was markedly inhibited by CREB inhibitor. In ALS models, the PAK4/CREB pathway was inhibited, and cell apoptosis increased. In vivo experiments revealed that PAK4 overexpression in the spinal neurons of hSOD1^G93A^ mice suppressed MN degeneration, prolonged survival and promoted the CREB pathway.

**Conclusions:**

PAK4 protects MN from degeneration by activating the anti‐apoptotic effects of CREB signalling, suggesting it may be a therapeutic target in ALS.

## Graphical abstract

Schematic representation of the mechanism of PAK4 protecting MN from apoptosis in ALS. PAK4 increases CREB levels and activation, leading to the upregulation of PGC‐1a and Bcl‐2, thereby decreasing cleaved‐caspase3 levels, and inhibiting MN degeneration. miR‐9‐5p is responsible for the decreased expression of PAK4 in ALS.

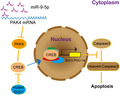

## 1 INTRODUCTION

Amyotrophic lateral sclerosis (ALS) is a heterogeneous, fatal neurodegenerative disorder characterized by progressive loss of MN in the brain, brainstem and spinal cord, leading to motor and extra‐motor symptoms.^1^, ^2^, ^3^ Approximately 5%‐10% of patients with ALS are familial (fALS), most often in an autosomal dominant pattern,^4^, ^5^ whereas the rest of cases are sporadic (sALS) and may result from genetic mutations or environmental exposure.^6^ Mutations in the SOD1 gene account for ~20% of fALS and ~5% of sALS.^7^, ^8^, ^9^ Transgenic mice carrying mutant SOD1 develop progressive paralysis^10^ and exhibit the complex pathophysiology of ALS disease, which have been widely used in the research of ALS aetiology and treatment.^11^ The exact mechanism underlying MN degeneration in ALS is still mostly unknown. Currently, only riluzole and edaravone are approved by FDA for ALS clinical therapy. However, they could provide limited efficacy.^12^, ^13^ Thus, strategies to promote MN survival remain an urgent need for ALS treatment.

cAMP‐response‐element binding protein (CREB) is a crucial transcription factor in the brain that regulates essential physiological functions including neuronal excitability, dendritic growth, long‐term synaptic plasticity formation and neuronal survival.^14^, ^15^, ^16^, ^17^, ^18^, ^19^, ^20^ CREB mediates neuroprotection mainly via its downstream genes, such as peroxisome proliferator‐activated receptor gamma coactivator‐1 alpha (PGC‐1a),^21^ brain‐derived neurotrophic factor^22^ and B‐cell lymphoma 2 (Bcl‐2).^23^ Deregulation of CREB signalling is associated with several neurodegenerative diseases, for instance, Alzheimer's disease (AD), Parkinson's disease (PD), Huntington's disease (HD) and ALS.^22^, ^24^, ^25^, ^26^ Restoring CREB activation rescues defects in dendrite morphogenesis in TDP‐43 misregulated neurons.^27^ In particular, human adipose stem cell extract treatment improves MN function and prolongs the life span in ALS mice by increasing the expression of pCREB.^28^ Moreover, our group has demonstrated that the neuroprotective effect of lithium for ALS relies on attenuating the improper binding of mutant SOD1 protein aggregates with pCREB.^29^ Although CREB signal transduction networks are complex and obscure, it has been proved that the CREB pathway plays a neuroprotective role in ALS disease.

The serine/threonine p21‐activated kinases (PAKs) are comprised of six mammalian proteins that are divided into group I (PAK1 to PAK3) and group II (PAK4 to PAK6) in terms of their structural and functional features.^30^ Group II PAKs contribute to a wide range of intracellular processes including cytoskeletal dynamics, cell growth, tumorigenesis, neuronal dysfunction and cell survival.^30^, ^31^, ^32^, ^33^, ^34^ Studies on PAK4 have revealed its pivotal role in cytoskeletal remodelling, neuronal development, axonal outgrowth and neuronal survival.^24^, ^35^, ^36^ PAK4 knockout mice display dramatic defects in the heart and nervous system, with striking abnormalities observed in axonal outgrowth and neural tube formation.^36^ Studies have shown that PAK4 expression in PD patients is downregulated and that overexpressing it promotes DA neurons survival in the a‐synuclein rat model.^24^ Deregulation of AKT signalling is involved in ALS. Restoring AKT activation rescues MN from death in ALS.^37^, ^38^, ^39^, ^40^ PAK4 is linked to AKT activity^41^, ^42^; thus, PAK4 may promote the survival of MN through AKT. Together, these studies suggest that PAK4 plays a pivotal role in neuronal pathophysiology.

PAK4 has been shown to increase the levels and activation of CREB globally in diverse cell types.^24^, ^43^, ^44^, ^45^ Given that PAK4 targets CREB and that CREB promotes MN survival, maintaining PAK4 activation is expected to play a pivotal role in preventing the progressive loss of MN in ALS. Therefore, we hypothesized that decreased PAK4 activation might involve in the pathogenesis of ALS. In this study, we found that the expression and activation of PAK4 were deregulated in ALS models. Overexpression of PAK4 protected MN from hSOD1^G93A^‐induced apoptosis, and this neuroprotective effect was alleviated by CREB inhibitor. Finally, hSOD1^G93A^ mice injected with LV‐PAK4 manifested a delay in disease onset, prolongation of survival and promotion of CREB signalling. These data suggest that the neuroprotective role of PAK4 in ALS MN is mediated by the CREB pathway.

## 2 METHODS

### 2.1 Animals

Transgenic mice carrying the hSOD1^G93A^ gene on the C57BL/6J strain background were obtained from Jackson Laboratory (Bar Harbor, ME, USA). All mice were bred and maintained on a 12 hours day/night cycle with adequate food and water. The offspring were identified by polymerase chain reaction (PCR) analysis of tail DNA.^46^ Transgenic female mice were culled at postnatal day 75, 120 and 140, corresponding to presymptomatic, early‐sym stage and late‐sym stage of the disease. Age‐matched non‐transgenic female littermates were used as wild‐type controls. All animal experiments were performed in accordance with the International Animal Care Guidelines and were approved by the Experimental Animal Ethics Committee of Harbin Medical University.

### 2.2 Generation of a hSOD1G93A‐transfected NSC34 cell line and cell cultures

The mouse neuroblastoma × spinal cord NSC34 cell line was a gift from Cedarlane Laboratories (Vancouver, Canada). NSC34 cells were stably transfected with hSOD1^G93A^ (mSOD1), human wild‐type SOD1 (wtSOD1) or an empty lentivirus vector (pLV) separately as described.^47^ Puromycin at 200 μg/mL (G418, Invitrogen) was used to maintain a stable cell line translation. Cells were cultivated at 37ºC in a 5% CO_2_‐atmosphere in DMEM‐supplemented 10% foetal bovine serum (FBS, Natocor) and 100 U/mL penicillin‐streptomycin.

### 2.3 Immunohistochemistry, immunofluorescence and analysis

After anaesthesia, the hSOD1^G93A^ and age‐matched non‐transgenic female littermates mice underwent transcardial perfusion with PBS, followed by perfusion with 4% PFA (paraformaldehyde; PH = 7.4). The spinal cords were dissected and fixed in 4% PFA for 24 hours, and then were embedded in paraffin. 3 μm sections of cervical or lumbar enlargements were prepared for immunohistochemistry and immunofluorescence staining. In brief, after deparaffinization and dehydration, sections were incubated with a primary antibody against PAK4 (1:200, Santa Cruz Biotechnology) overnight at 4°C. For the visualization, sections were incubated with secondary antibodies followed by DAB (3,3'‐diaminobenzidine) staining. The images were captured using a Leica microscope (Leica, Wetzlar, Germany) and analysed by Image‐Pro Plus 6.0 software (Media Cybermetics, Inc, Silver Spring, MD, USA). For immunofluorescence staining, sections were incubated with primary antibody against PAK4 (1:150, Santa Cruz Biotechnology), anti‐MAP‐2 antibody (1:200, Bioss Biotechnology Co) and anti‐cherry (1:500, Abcam) at 4°C overnight. After washing, secondary anti‐mouse/rabbit antibodies (1:200, ZSGB‐BIO) were used. Next, the sections were incubated with four, 6‐diamidino‐2‐phenylindole (DAPI; Beyotime) for 5 minutes at room temperature. The images were captured by an inverted fluorescence microscope (Zeiss Vert A1, Germany). At least eight images (20×) were taken in the right and left ventral horn (VH) for each mouse. Cells with ≥20 μm in maximum diameter in the VH were included for MN immunoreactivity analysis and MN quantification.^38^

### 2.4 Immunofluorescence cytochemistry analysis

Cultured cells were washed three times with PBS and fixed for 20 minutes in 4% PFA (PH = 7.4) at room temperature. After being penetrated with 0.2% Triton X‐100, cells were blocked with 5% BSA for 1 hour. Then, the cells were incubated overnight at 4°C with the primary antibodies (anti‐PAK4, Santa Cruz Biotechnology) at 1:150 dilutions. Following three washes in PBS, cells were incubated with FITC‐conjugated goat anti‐mouse IgG (1:100) for 2 hours at room temperature. After washing, cells were stained with DAPI for 3 minutes. An inverted fluorescence microscope was used for cell observation.

### 2.5 Western blotting analysis

The whole spinal cords from mice or prepared cells were lysed in RIPA lysis buffer (Beyotime Biotechnology Co., Ltd.) containing phosphatase and protease inhibitor mixture (Roche, 4906845001 and 04693132001). Lysates were centrifuged at 18630 *g* for 20 minutes at 4°C, and only the supernatants were analysed by Western blotting, as described previously.^47^ Primary antibodies: rabbit anti‐PAK4 (1:1000, Cell Signaling Technology), rabbit anti‐pPAK4 at Ser474 (1:1000, Cell Signaling Technology), rabbit anti‐CREB (1:1000, Cell Signaling Technology), rabbit anti‐pCREB at Ser133 (1:1000, Cell Signaling Technology), mouse anti‐Bcl‐2 monoclonal antibody (1:500, Bioss Antibodies), rabbit anti‐PGC‐1a (1:1000, Abcam), rabbit anti‐total caspase3 (1:2000, Abcam), rabbit anti‐cleaved caspase3 (1:500, Abcam) and mouse anti‐β‐actin (1:1000, Beyotime). The blots were incubated with Alexa Fluor‐800‐conjugated secondary antibody (Goat anti‐mouse or anti‐rabbit, 1:10 000, Li‐COR) at room temperature for 1 hour. For signal detection, an Odyssey infrared imaging system (Li‐COR, USA) was used according to the manufacturer's instructions. The density of the blots was determined using Image J (http://imagej.nih.gov/ij/).

### 2.6 qRT‐PCR

For PAK4 mRNA quantification, total RNA was extracted from spinal cords or prepared cells using Trizol (Cwbio); cDNA was generated using ReverTra Ace qPCR RT Master Mix with gDNA Remover (TOYOBO CO., LTD. Life Science Department OSAKA JAPAN). For miR‐9‐5p detection, total RNA was reverse‐transcribed using an miRNA cDNA Synthesis Kit (Cwbio). qRT‐PCR was performed on a Light Cycler 480 (Roche) using THUNDERBIRD SYBR qPCR Mix (TOYOBO CO.,LTD. Life Science Department OSAKA JAPAN) and an miRNA qPCR Assay Kit (Cwbio) according to the manufacturer's protocol. The following mouse primers were used: mPAK4, forward primer: 5′‐GACTACCGGCACGAGAACG‐3', reverse primer: 5′‐CATCATGGGTCAGCAAGATAGAG‐3'; mβ‐actin, forward primer: 5′‐CCAGCCTTCCTTCTTGGGTAT‐3', reverse primer: 5′‐TGCTGGAAGGTGGACAGTG AG‐3'; miR‐9‐5p, forward primer: 5′‐ACACTCCAGCTGGGAGTATGTCGATCTATTG‐3′, reverse primer: 5′‐TGG TGTCGTGGAGTCG‐3′; U6, forward primer: 5′‐CTCGCTTCGGC AGCACA‐3′, reverse primer: 5′‐AACGCTTCACGAATTTGCGT‐3′. The relative expression of PAK4 mRNA and miR‐9‐5p was analysed using the 2^−ΔΔCT^ method. qRT‐PCR reactions were carried out in three independent runs in triplicate.

### 2.7 Oligonucleotide transfection

The siRNA sequences specific against mouse PAK4 (sense: 5′‐GGAUGAACGAGGAA CAGAUTT‐3', antisense: 5′‐AUCUGUUCCUCGUUCAUCCTT‐3') and non‐targeting siRN‐ A control sequences (sense: 5′‐UUCUCCGAACGUGUCACGUTT‐3'; antisense: 5′‐ACGU‐ GACACGUUCGGAGAATT‐3') were obtained from Gene Pharma Co., Ltd (Shanghai, China). The miR‐9‐5p inhibitor and miR‐NC were chemically synthesized by Gene Pharma. All transfections were carried out according to the manufacturer's protocols. Briefly, NSC34 cells were seeded in a 24‐well format, and after reaching 70%‐80% confluence, the siRNAs and miRNA inhibitor were transfected into cells using Lipofectamine 2000 (Invitrogen). The effectiveness of transfection was evaluated by qRT‐PCR and Western blotting.

### 2.8 Plasmid transfection and treatment

The cDNA plasmid (OriGene, Beijing, China) mediated overexpression of PAK4 in mSOD1 cells was performed as previous description^48^ using Lipofectamine 2000. mSOD1 cells transfected with p3xFlag‐CMV‐14 construct served as controls. At 48 hours after transfection, cells containing cDNA or vehicle control were incubated with 50.26 ng/mL CREB inhibitor (compound 3i, Selleckchem, US). Then, mSOD1 cells were divided into four groups: (a) cDNA‐PAK4 transfection group (PAK4^+^); (b) empty‐vector transfection group (EV); (c) empty‐vector transfection plus the CREB inhibitor group (EV + 3i); and (d) cDNA‐PAK4 transfection plus the CREB inhibitor group (PAK4^+^ + 3i). All groups were incubated under standard growth conditions.

### 2.9 Cell apoptosis assay

To detect apoptosis, at 72 hours after siPAK4 transfection or 3i treatment, cultures were subjected to flow cytometric analysis using an Annexin V‐FITC/PI Kit (4A Biotech Co, Ltd) and TdT‐mediated dUTP nick end labelling (TUNEL) staining using an In Situ Cell Death Detection Kit (Roche, Basel, Switzerland). In brief, the prepared cells were stained with Annexin V‐FITC for 5 minutes in the dark before being analysed by flow cytometry. TUNEL staining was performed according to the manufacturer's instructions. The cells were incubated with TdT enzyme for 1 hour at 37°C in a humid chamber and then counter‐stained with DAPI. The apoptosis index was determined by the number of TUNEL‐positive/total number of nucleated cells × 100%.

### 2.10 Injections

The lentivirus vector that carried PAK4 cDNA (LV‐PAK4) and the lentivirus control (LV‐cherry) were purchased from the GenePharma Corporation. 60 days of age female hSOD1^G93A^ mice were randomly divided into two groups: hSOD1^G93A^/LV‐PAK4 and hSOD1^G93A^/LV‐cherry groups (n = 15/group), and they were injected with LV‐PAK4 or LV‐cherry (6 µL, 2 × 10^8^ TU/mL) into the mouse's L5 and L6 subarachnoid space using a glass micro‐needle.

### 2.11 Disease course analysis and behaviour tests

To test motor function, rotarod performance was examined in LV‐PAK4 or LV‐cherry injected hSOD1^G93A^ mice starting at 80 days of age. After 1 week of training, each mouse was placed on the rotating rod at 16 rpm every 2 days to record the latency time before the mouse would fall off. The test was repeated three times for each animal. Disease onset was defined as the age at which a mouse could no longer stay on the rotating rod for 180 seconds. The date of disease endpoint was recorded when the mouse could not rise within 30 seconds after being placed on its side.

### 2.12 Statistical analyses

The data were analysed using SPSS version 23.0 and GraphPad Prism 8.0 software (GraphPad Software, USA). Statistical significance was evaluated with Student's *t*‐test or one‐way analysis of variance (ANOVA). Quantitative data were expressed as mean values of at least three determinations ± SD. Tests were considered statistically significant at *P* < .05.

## 3 RESULTS

### 3.1 Reduced PAK4 expression in the spinal cord of hSOD1G93A mice

**FIGURE 1:**
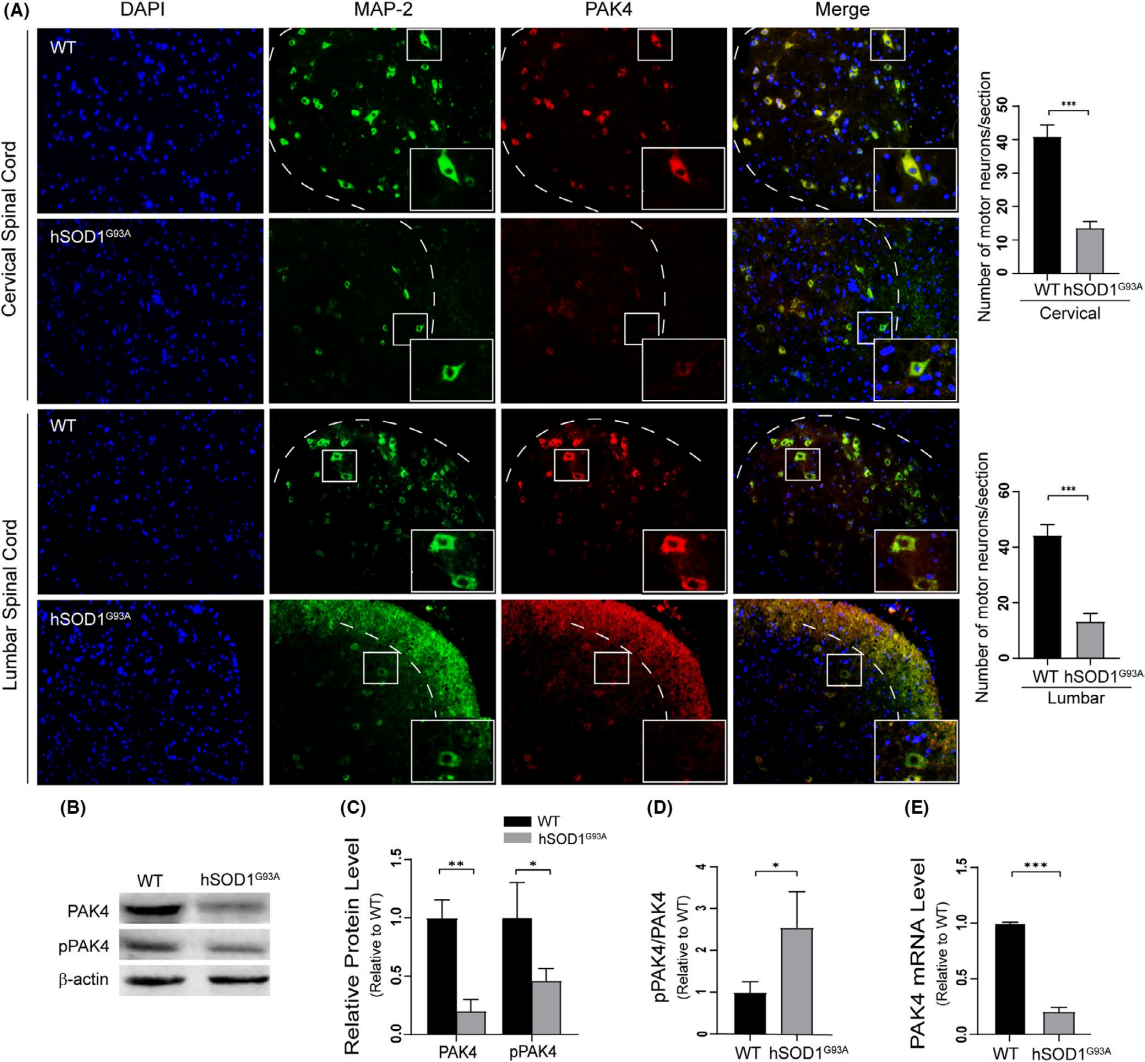
Decreased PAK4 expression and activation in motor neuron of hSOD1^G93A^ mice. A, Confocal microscopy images showed that PAK4 expression in MN of p140 hSOD1^G93A^ mice was lower than that of non‐transgenic littermates (WT), n = 3/group. The number of MN surviving in hSOD1^G93A^ mice was significantly decreased compared with WT (cervical spinal cord: 41.00 ± 3.61 vs 13.67 ± 2.08; lumbar spinal cord: 44.33 ± 4.04 vs. 13.33 ± 3.05; *P* < .05). B, Immunoblotting of spinal cord extract from p140 hSOD1^G93A^ mice and WT was conducted to detect PAK4 and pPAK4 expression. C, Quantification of PAK4 and pPAK4 blots in (B) normalized to β‐actin. Relative PAK4 and pPAK4 protein levels in hSOD1^G93A^ mice were significantly downregulated. D, pPAK4/PAK4 ratio in spinal cords of hSOD1^G93A^ mice was high. E, qRT‐PCR revealed that hSOD1^G93A^ mice showed remarkably decreased PAK4 mRNA levels (n = 6/group). Data were shown as means ± SD. Student's *t*‐test was used to evaluate statistical significance, *P < .05, **P < .01, ***P < .001

Immunofluorescence analyses of the spinal cords of hSOD1^G93A^ mice and non‐transgenic littermates (WT) were performed to detect differences in PAK4 levels associated with ALS. Results showed that for hSOD1^G93A^ mice, the number of MN (green) was significantly reduced, and MN exhibited a weaker fluoresence of PAK4 (red) compared with those of WT (Figure 1A). Decreased PAK4 expression in ALS was confirmed by Western blotting. We also found that the protein levels of phospho‐PAK4^s474^, an index of PAK4 activation, were lower in p140 hSOD1^G93A^ mice compared to WT (Figure 1B and C). The pPAK4/PAK4 ratio was greater in hSOD1^G93A^ mice (Figure 1D). These data suggested that the expression and activation of PAK4 in hSOD1^G93A^ mice were both downregulated. Next, we analysed PAK4 mRNA levels in the spinal cords of hSOD1^G93A^ mice (n = 6) and WT (n = 6) using qRT‐PCR. It was found that the gene‐expression pattern was followed by protein expression. The levels of PAK4 mRNA were significantly reduced in hSOD1^G93A^ mice (Figure 1E).

**FIGURE 2:**
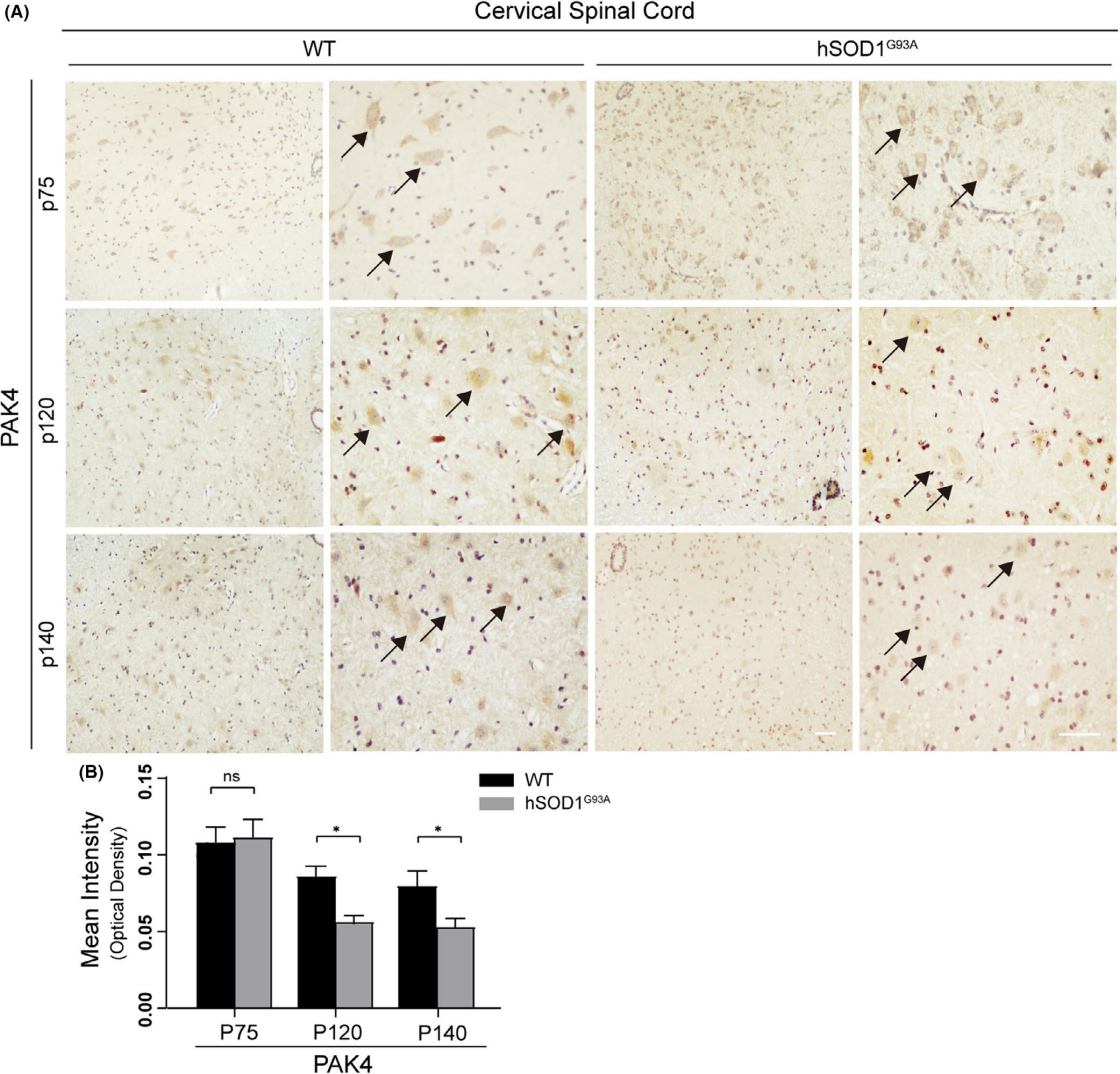
The expression of PAK4 decreased in the cervical spinal cords of hSOD1^G93A^ mice during disease progression. Paraffin sections of cervical spinal cords from hSOD1^G93A^ mice and WT at three stages of the disease were subjected to immunohistochemistry. A, PAK4‐stained MN (maximum diameter ≥ 20 μm) were detected in the anterior horn of spinal cords from both hSOD1^G93A^ mice and WT. Scale bar = 50 μm. B, At stages p120 and p140, OD values of PAK4 significantly decreased in hSOD1^G93A^ mice, while there was no deregulation at stage p75 (n = 3/group, six sections/mouse). Data were provided as means ± SD and were tested for significance using Student's *t*‐test. ns ≥ .05, *P < .05

To characterize the temporal expression patterns of PAK4 in ALS mice, we evaluated PAK4 levels in hSOD1^G93A^ and WT mice at three stages of the disease (the presymptomatic stage P75, early‐sym stage P120 and late‐sym stage P140) using immunohistochemistry. Results showed that compared with WT, PAK4 immunoreactivity was significantly lower at early‐sym and late‐sym stages of the disease in ALS mice (Figure 2A and Figure S1A). However, at the presymptomatic stage, there was no statistical difference in the optical density (OD) values of PAK4 between hSOD1^G93A^ mice and WT (Figure 2B and Figure S1B). The levels of PAK4 at three stages of the disease were further confirmed by Western blotting (Figure 1B and Figure S2). Taking these data together, we predicted that PAK4 was involved in the pathophysiological process of ALS.

### 3.2 PAK4 protein levels are downregulated in mSOD1 cells

**FIGURE 3:**
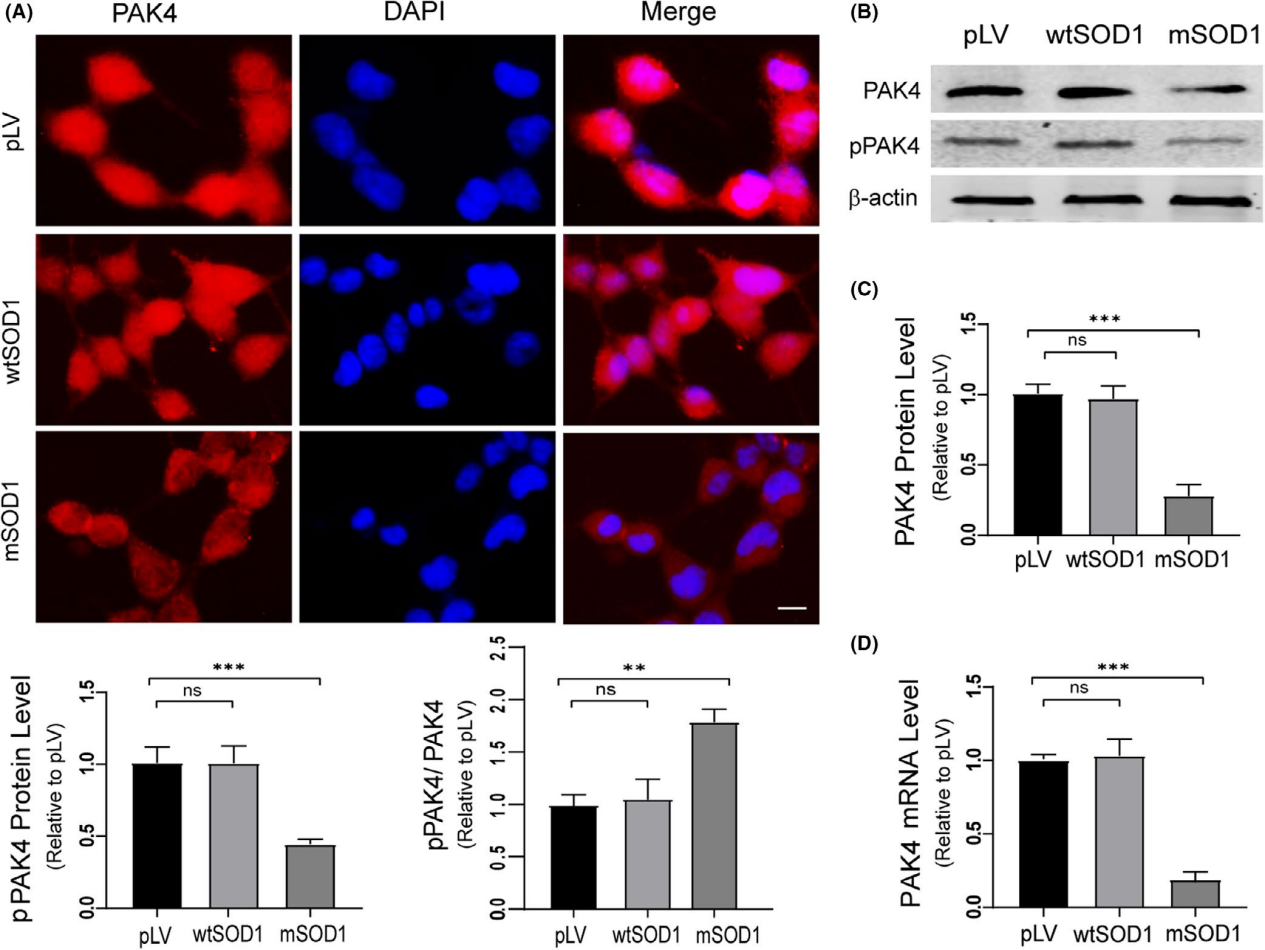
Reduced expression of PAK4 is identified in mSOD1 cells. A, Confocal microscopy images of PAK4 (red) in pLV, wtSOD1 and mSOD1 cells, respectively. The weakest intensity of PAK4 staining was observed in mSOD1 cells, while similar intensities were seen in wtSOD1 and pLV cells. Scale bar = 50 μm. B, Immunoblotting analysis of PAK4 and pPAK4 protein expression levels was conducted with lysates from control pLV, wtSOD1 and mSOD1 cells. C, Quantification of PAK4 and pPAK4 blots in (B) normalized to β‐actin. Relative PAK4 and pPAK4 protein levels were significantly decreased in mSOD1 cells. pPAK4/PAK4 ratio was highest in mSOD1 cells. D, qRT‐PCR analysis detected the mRNA expression of PAK4. For relative quantification of PAK4 mRNA expression, the 2^−ΔΔCt^ method was conducted using β‐actin for normalization; data were provided as means ± SD. ANOVA with Student‐Newman‐Keuls post hoc analysis was used to evaluate statistical significance, ns ≥ .05, *P < .05, **P < .01, ***P < .001

Next, we explored whether the expression and activation of PAK4 were altered in vitro models of ALS. NSC34 cells exhibit many MN characteristics, including the generation of action potentials, expression of neurofilament proteins and synthesis of acetylcholine.^49^, ^50^, ^51^ Our group has confirmed that choline acetyltransferase, an MN marker, is expressed in NSC34 cells.^40^ Thus, NSC34 cells stably transfected with hSOD1^G93A^ (mSOD1) were used as a cell model of ALS in subsequent studies. Immunocytochemistry staining showed that compared to pLV cells, mSOD1 cells displayed weaker fluorescence intensity of PAK4, while wtSOD1 cells displayed a similar intensity (Figure 3A). Decreased PAK4 expression in mSOD1 cells was confirmed by Western blotting. We also found that compared with pLV, protein levels of pPAK4 were significantly decreased in mSOD1 cells, while wtSOD1 and pLV cells had similar pPAK4 protein levels (Figure 3B and C). The pPAK4/PAK4 ratio in mSOD1 cells was greater than in control pLV and wtSOD1 cells (Figure 3C). These data suggested that the expression and activation of PAK4 in mSOD1 were both downregulated. Next, qRT‐PCR analysis suggested that PAK4 mRNA levels of mSOD1 cells were robustly downregulated to approximately 18.9% compared to pLV cells. PAK4 mRNA expression in wtSOD1 and pLV cells showed no statistical difference (Figure 3D).

### 3.3 Knockdown of PAK4 promotes motor neuron degeneration through inhibiting CREB pathway

**FIGURE 4:**
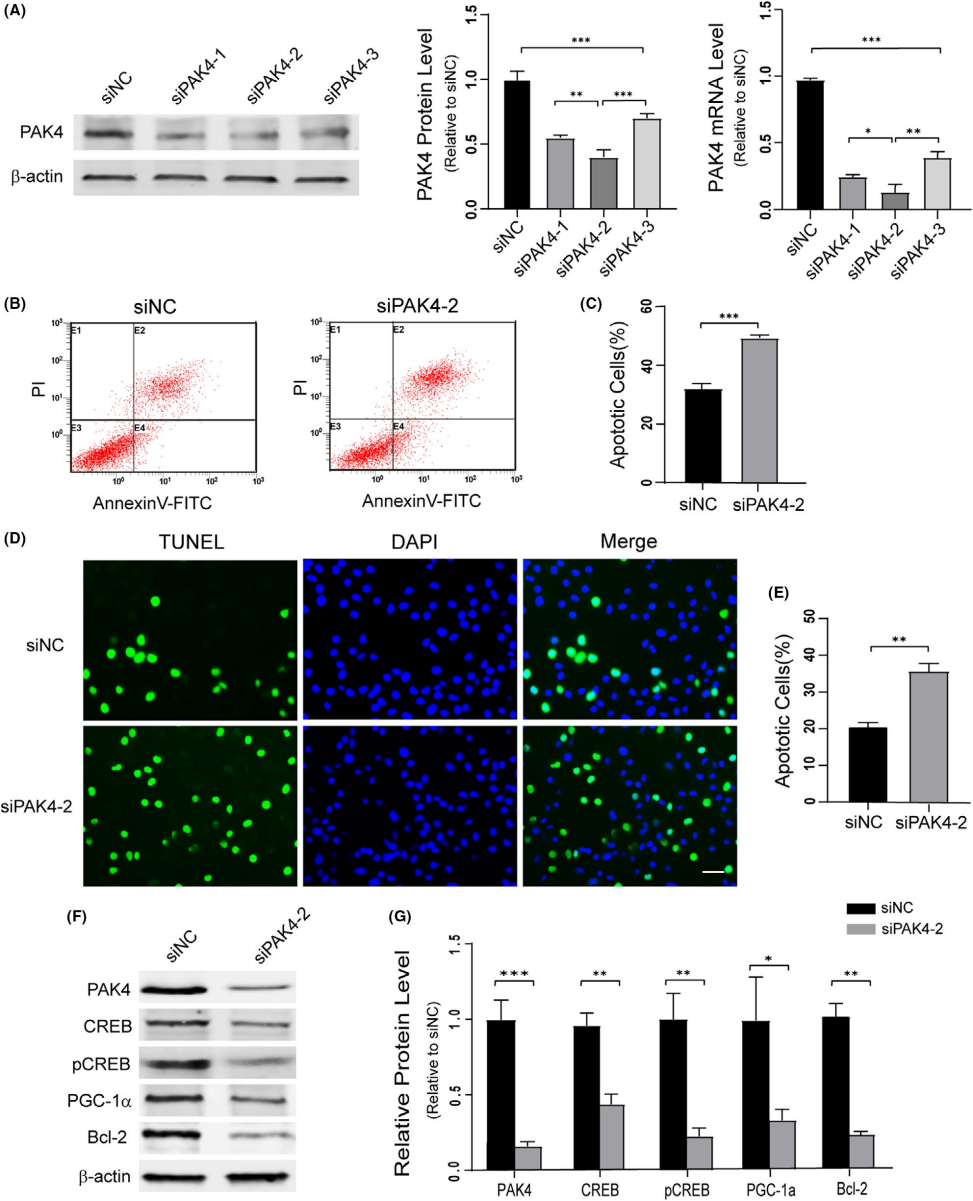
Knockdown of PAK4 triggers neurodegeneration and inhibits CREB pathway. A, Western blotting analysis of the efficiency of PAK4 knockdown using siRNAs. The mRNA level of PAK4 was detected by qRT‐PCR in NSC34 cells transfected with siNC or siPAK4. B, Flow cytometry detected the apoptosis of NSC34 cells after transfection with siPAK4‐2 or siNC for 72 h. C, Quantification of cell apoptosis. D and E, TUNEL staining of NSC34 cells after treatment with siPAK4‐2. F, Western blotting of PAK4/CREB pathway signalling proteins expression levels in PAK4 knockdown NSC34 cells. G, Quantification of the blots in (F) normalized to β‐actin. Data were presented as means ± SD. *P < .05, **P < .01, ***P < .001. Student's *t*‐test (C, E, G) or ANOVA with Student‐Newman‐Keuls post hoc analysis (A). Scale bar = 50 μm

To determine the biological role of PAK4 in MN, we selected NSC34 cells with many MN characteristics for further study. Three siRNAs targeting PAK4 were transfected into NSC34 cells, and the efficiency of PAK4 knockdown was evaluated by Western blotting and qRT‐PCR (Figure 4A). We selected siPAK4‐2 for subsequent experiments because of its obvious knockdown effects. Flow cytometry analysis suggested that NSC34 cells transfected with siPAK4‐2 yielded a significantly higher rate of cell apoptotic death (49.34 ± 0.95%) than siNC control (32.07 ± 2.45%; Figure 4B and C). Consistently, TUNEL staining indicated that knockdown of PAK4 significantly increased the percentage of cells experiencing apoptotic death (35.67 ± 2.51%), and in the siNC group, the percentage of cell apoptosis was 20.57 ± 1.50% (Figure 4D and E). These data suggested that PAK4 protected NSC34 cells from apoptotic death.

Next, we further investigated the mechanism by which PAK4 promoted cell survival. Previous studies have revealed that PAK4 is a critical regulator of CREB and that CREB promotes MN survival.^52^ Thus, Western blotting of PAK4, CREB, pCREB and the CREB target proteins (PGC‐1a and Bcl‐2) was performed with extract from siNC and siPAK4‐2 groups. The results showed that the levels of CREB, pCREB, Bcl‐2 and PGC‐1a were decreased in PAK4 knockdown NSC34 cells. These data indicated that PAK4 might inhibit MN degeneration via the CREB pathway (Figure 4F and G).

### 3.4 CREB pathway mediates PAK4‐induced neuroprotection in mSOD1 cells

**FIGURE 5:**
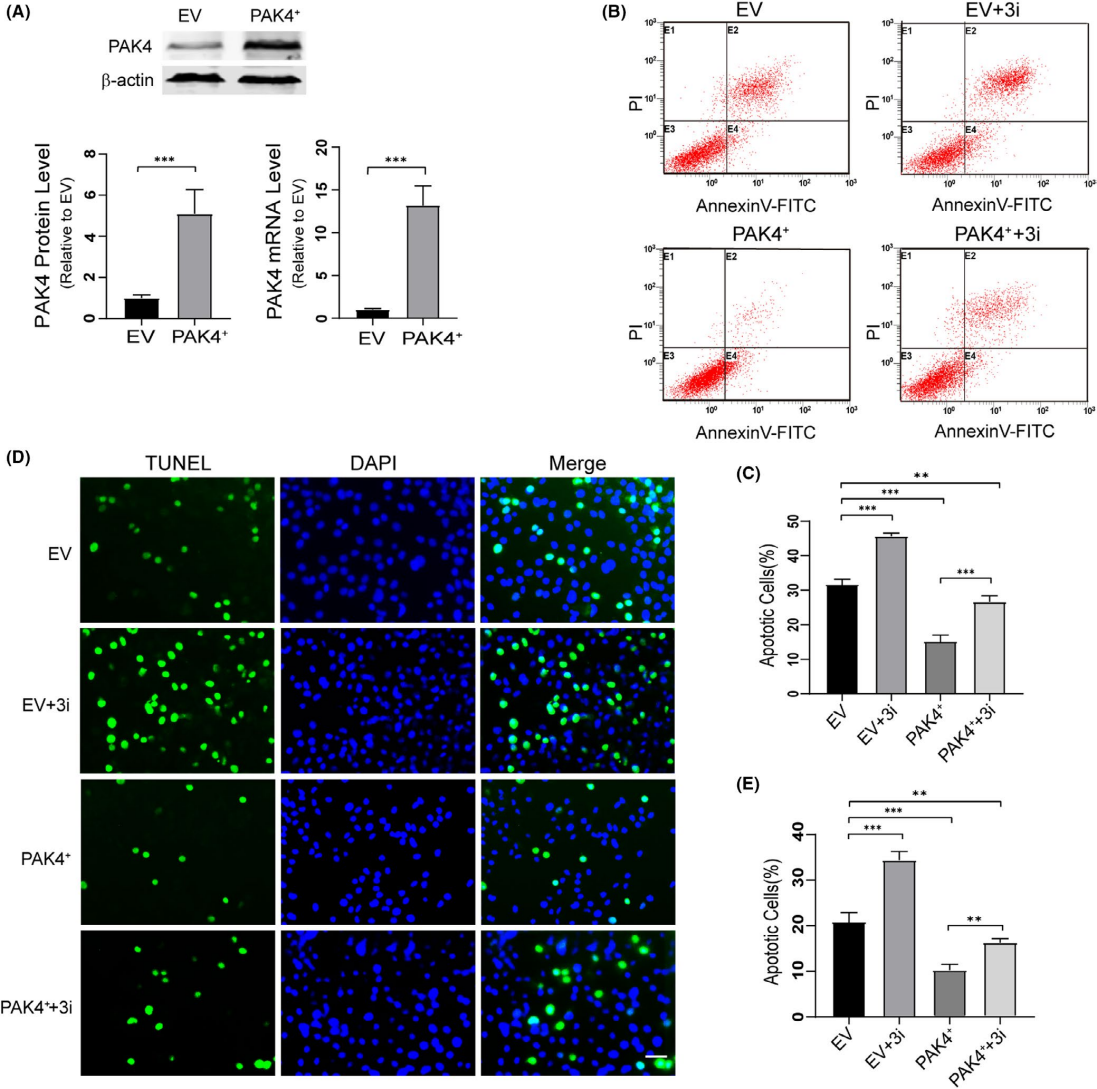
Overexpression of PAK4 protects motor neurons from hSOD1^G93A^ induced apoptosis. A, The effectiveness of PAK4 plasmid transfection was evaluated by qRT‐PCR and Western blotting, and β‐actin served as a loading control. B and C, 48 h after transfection, mSOD1 cells were incubated with 3i (50.26 ng/mL), and cell apoptosis was determined by flow cytometry. D, The mSOD1 cells transfected with PAK4 plasmid or EV were treated with 3i (50.26 ng/mL) for 72 h. Then, cell apoptosis was determined by TUNEL staining. Scale bar = 50 μm. E, Quantification analysis of the TUNEL staining. Data from the experiments were presented as means ± SD. *P < .05, **P < .01, ***P < .001, vs Control group. ANOVA with Student‐Newman‐ Keuls post hoc analysis (C, E) or Student's *t*‐test (A)

**FIGURE 6:**
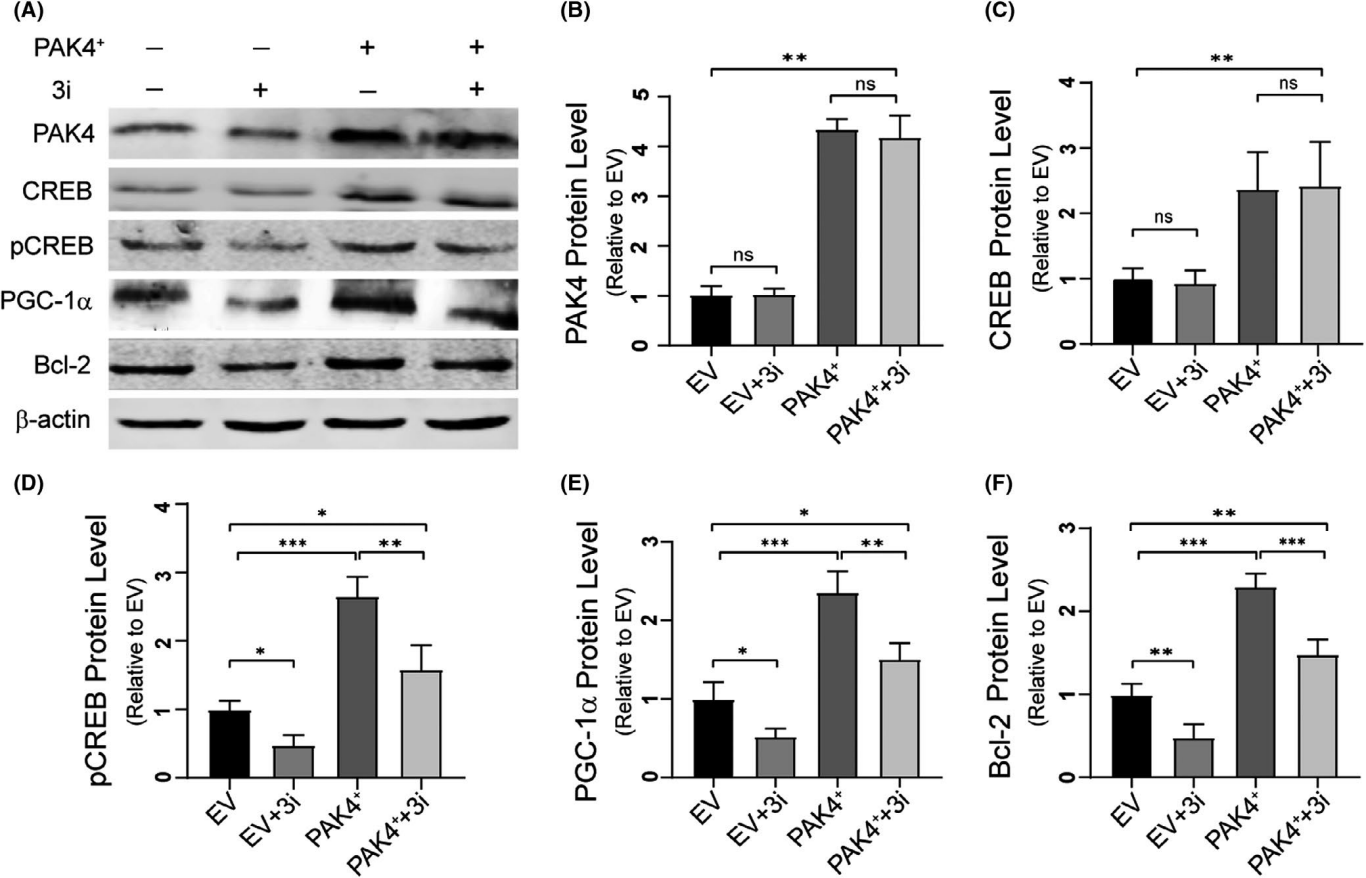
CREB pathway mediates PAK4‐induced neuroprotection. A, Immunoblotting of cell extract from EV, EV + 3i, PAK4^+^ and PAK4^+^+3i groups. B‐F, Quantitative analysis of PAK4 (B), CREB (C), pCREB (D), PGC‐1a (E) and Bcl‐2 (F) levels in (A) normalized to β‐actin. Data were presented as means ± SD. ns ≥ .05, *P < .05, **P < .01, ***P < .001, vs Control group. ANOVA with Student‐Newman‐Keuls post hoc analysis

To investigate whether the neuroprotective role of PAK4 would extend to cell models of ALS, we upregulated PAK4 expression using a plasmid vector for PAK4 in mSOD1 cells. The results of Western blotting and qRT‐PCR showed that PAK4 plasmid significantly increased PAK4 expression in mSOD1 cells (Figure 5A). To further determine whether the possible neuroprotective effect of PAK4 in mSOD1 cells was mediated by CREB signalling, we selectively inhibited CREB function by using compound 3i (a specific inhibitor of CREB) while transfecting the PAK4 plasmid or empty‐vector (EV). The results showed that the percentage of cell apoptotic death was lowest in the PAK4^+^ group (15.19 ± 1.98%). In the PAK4^+^+3i group, 3i impaired the neuroprotective function of PAK4 (24.93 ± 2.27%). In the EV and EV + 3i groups, the percentages of apoptotic death were 31.70 ± 1.68% and 45.70 ± 0.87% (Figure 5B and C). This effect was further confirmed by TUNEL (Figure 5D and E), and administration of PAK4 improved mSOD1 cell viability (10.25 ± 1.29%). The ability of PAK4 to relieve mSOD1‐induced cytotoxicity was compromised in the PAK4^+^+3i group (16.28 ± 0.86%). In the EV and EV + 3i groups, the percentages of apoptotic death were 20.84 ± 2.17% and 34.39 ± 1.89%. Furthermore, Western blotting studies confirmed increased PAK4, CREB, pCREB, Bcl‐2 and PGC‐1a levels in the PAK4^+^ group compared with EV control (Figure 6A‐F). 3i inhibited the phosphorylation and function of CREB effectively. Compared with the PAK4^+^ group, the levels of pCREB, Bcl‐2 and PGC‐1a were decreased in the PAK4^+^+3i group, while the levels of PAK4 and CREB showed no significant changes (Figure 6A‐F). Thus, these data suggested that CREB and its target proteins BCL‐2, PGC‐1a mediated the neuroprotective effect of PAK4.

### 3.5 PAK4 deficiency increases motor neuron degeneration by inhibiting CREB pathway in mSOD1 cells and hSOD1G93A mice

**FIGURE 7:**
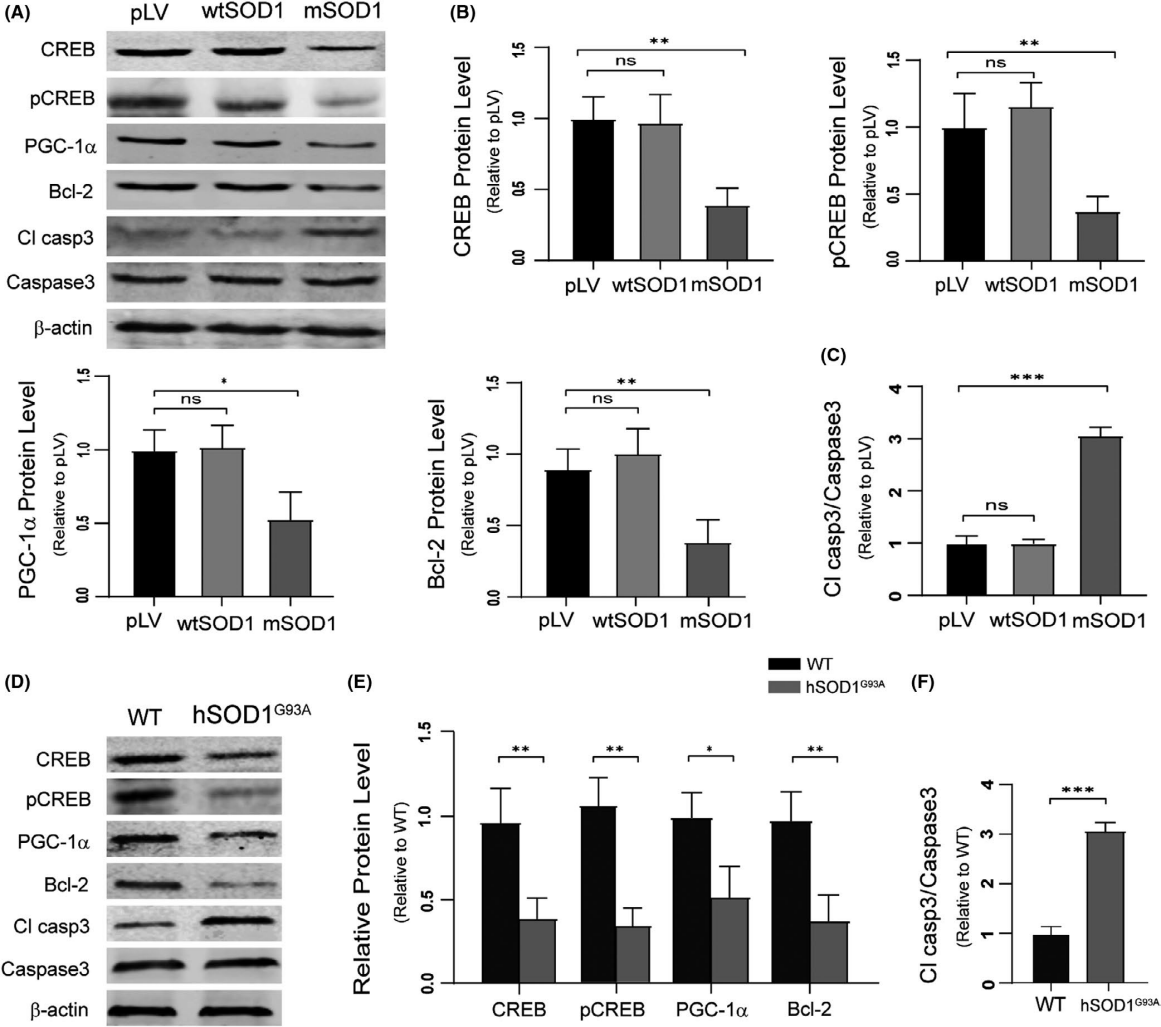
Low levels of PAK4 promote motor neuron degeneration through suppressing the CREB pathway in ALS in vitro and in vivo models. A, Western blotting analysis of CREB, pCREB, PGC‐1a, Bcl‐2, caspase3 and cleaved‐caspase3 levels was conducted with lysates from pLV, wtSOD1 and mSOD1 cells. B and C, Quantification analysis of CREB, pCREB, PGC‐1a, Bcl‐2 and cleaved‐caspase3 in (A) normalized to β‐actin and caspase3. D, CREB, pCREB, PGC‐1a, Bcl‐2, caspase3 and cleaved‐caspase3 levels of the spinal cord lysates from WT and hSOD1^G93A^ mice were analysed by Western blotting. E and F, Quantification of CREB, pCREB, PGC‐1a, Bcl‐2 and cleaved‐caspase3 in (D) normalized to β‐actin and caspase3. Data were presented as means ± SD. ns ≥ .05, *P < .05, **P < .01, ***P < .001 vs Control group. Student's *t*‐test

To explore the effects of deregulated PAK4 expression on downstream consequences and neurodegeneration in ALS models, the protein levels of CREB signalling and cleaved‐caspase3 were analysed in mSOD1 cells and the spinal cords from hSOD1^G93A^ mice. Immunoblotting analysis revealed that mSOD1 cells exhibited low levels of CREB, pCREB and CREB target proteins than pLV cells, while wtSOD1 cells displayed similar levels (Figure 7A and B). Downregulated PAK4 markedly increased the levels of cleaved‐caspase3 in mSOD1 cells (Figure 7A and C). In the in vivo study, the levels of CREB, pCREB, Bcl‐2 and PGC‐1a in the spinal cords of late‐sym stage hSOD1^G93A^ mice were significantly reduced compared with WT (Figure 7D and E). Moreover, hSOD1^G93A^ mice exhibited higher levels of cleaved‐caspase3 than control (Figure 7D and F). These data suggested that downregulated PAK4 expression promoted neurodegeneration in ALS models by impairing the CREB pathway.

### 3.6 PAK4 is negatively regulated by miR‐9‐5p in ALS

**FIGURE 8:**
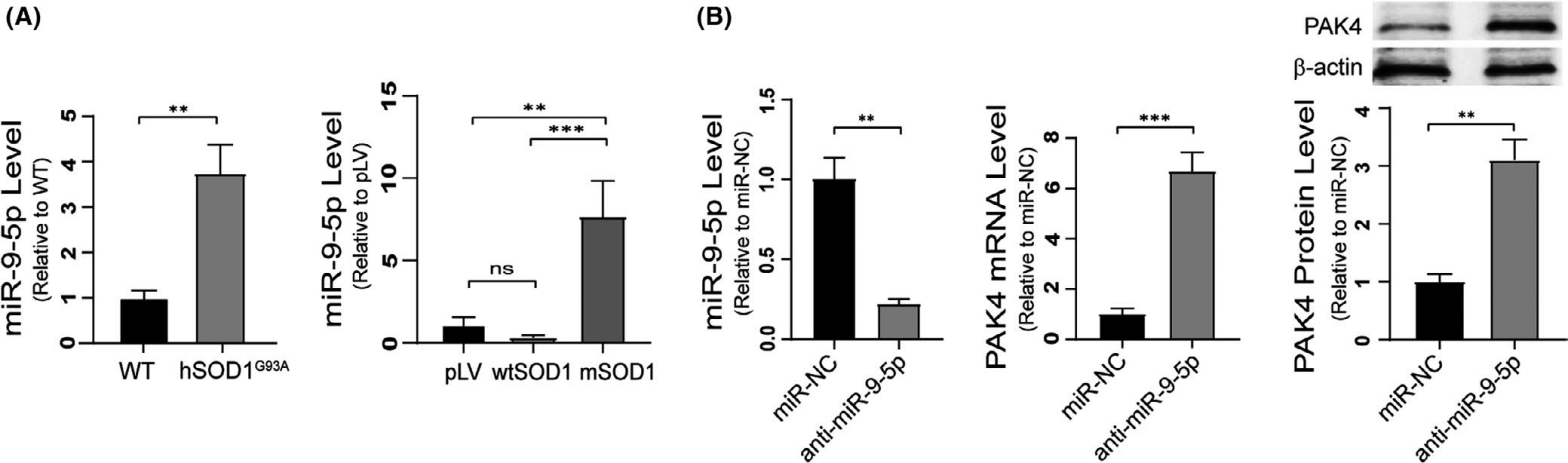
The expression of miR‐9‐5p increases in ALS and reversely regulates PAK4 mRNA levels. A, The expression levels of miR‐9‐5p in spinal cords from hSOD1^G93A^ mice and in mSOD1 cells were determined by qRT‐PCR. B, The expression of miR‐9‐5p and PAK4 at mRNA and protein levels in mSOD1 cells transfected with anti‐miR‐9‐5p or miR‐NC were determined by qRT‐PCR and Western blotting. Data were presented as means ± SD. *P < .05, **P < .01, ***P < .001. Statistical analyses were performed using Student's *t*‐test

MicroRNAs (miRNAs), small non‐coding RNAs, repress gene translation and promote target mRNA degradation.^53^ It has been reported that PAK4 is target regulated by a variety of miRNAs, including miR‐433,^54^ miR‐224,^55^ miR‐199a‐3p^56^ and miR‐9‐5p.^57^ Moreover, the expression of miR‐9‐5p in the cerebrospinal fluid of ALS patients is increased.^58^ We speculated that PAK4 might be inversely regulated by miR‐9‐5p in ALS. To test our hypothesis, we first analysed miR‐9‐5p levels in ALS vivo and vitro models using qRT‐PCR. Results showed that compared with control, the expression of miR‐9‐5p in spinal cords of p140 hSOD1^G93A^ mice was increased, especially in the mSOD1 cell line (Figure 8A). In studies, we have found that the mRNA levels of PAK4 were decreased in vivo and in vitro models of ALS (Figures 1E and 3D). To determine whether miR‐9‐5p negatively regulated PAK4 mRNA levels, miR‐9‐5p inhibitor transfection was conducted on mSOD1 cells. The results showed that downregulation of miR‐9‐5p significantly upregulated the expression of PAK4 mRNA and protein (Figure 8B).

### 3.7 Delayed disease onset and extended lifespan of hSOD1G93A mice injected with LV‐PAK4

**FIGURE 9:**
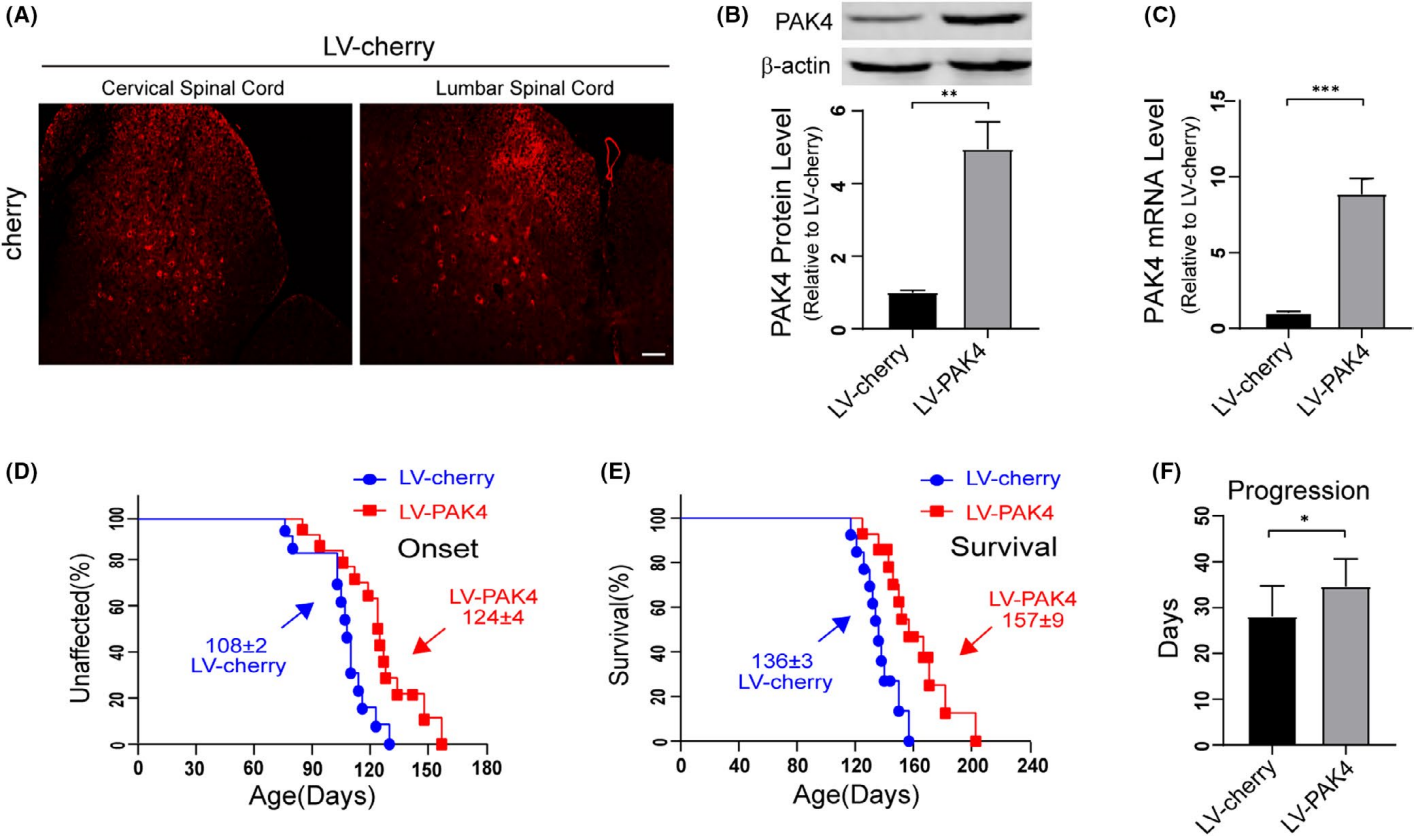
Overexpression of PAK4 in the spinal cord delays disease onset and increases the lifespan of hSOD1^G93A^ mice. A, Expression of cherry in the spinal cord of hSOD1^G93A^ mice injected with LV‐cherry (red) was examined using immunofluorescence. Scale bar = 50 μm. B, The protein expression levels of PAK4 were increased in spinal cord tissues of hSOD1^G93A^ mice injected with LV‐PAK4 compared with those of the hSOD1^G93A^ mice injected with LV‐cherry. C, The levels of PAK4 mRNA in the spinal cords of hSOD1^G93A^ mice injected with LV‐PAK4/LV‐cherry were analysed by qRT‐PCR. D‐F, LV‐PAK4 intraspinal injection into p60 hSOD1^G93A^ mice significantly postponed median disease onset (D) compared with LV‐cherry (LV‐PAK4:124 d; LV‐cherry:108 d; *P* < .01 ) and extended median survival (E) (LV‐PAK4: 157 d; LV‐cherry: 136 d; *P* < .01) and disease progression (F) (LV‐cherry: 28 d; LV‐PAK4: 35 d; *P* < .05). Data represent means ± SD. Statistical analyses were performed using Student's *t*‐test or Kaplan‐Meier survival analysis. *P < .05, **P < .01, ***P < .001

Since PAK4 played a neuroprotective role in vitro, we next evaluated the in vivo biological function of PAK4 in hSOD1^G93A^ mice. We injected hSOD1^G93A^ mice intraspinally at 60 days of age with LV‐PAK4 or LV‐cherry. Four weeks postinjection, the spinal cord sections of treated mice were examined for cherry expression by immunofluorescence (Figure 9A). The data showed that transduction efficiency of lentiviral vector was high in the ventral horns of the cervical and lumbar spinal cord. Western blotting and qRT‐PCR further determined the results. The protein levels of PAK4 were higher in the spinal cords of LV‐PAK4‐injected hSOD1^G93A^ mice compared with LV‐cherry‐injected ones (Figure 9B). Furthermore, qRT‐PCR detected significantly higher PAK4 mRNA levels in LV‐PAK4 injected hSOD1^G93A^ mice compared with the controls (Figure 9C).

Disease onset (measured by the rotarod test) was significantly delayed by a median of 16 d for hSOD1^G93A^ mice injected with LV‐PAK4 compared with the hSOD1^G93A^ littermates injected with LV‐cherry (LV‐PAK4:124 d; LV‐cherry:108 d; *P* < .01; Figure 9D). Moreover, survival of the LV‐PAK4‐injected hSOD1^G93A^ mice was remarkably extended, yielding a median survival time of 21 days beyond that of the LV‐cherry‐injected ones (LV‐PAK4:157 d; LV‐cherry:136 d; *P* < .01; Figure 9E). The LV‐PAK4 treated mice had increased disease duration (the time from disease onset to endpoint), indicating slower disease progression, compared with the LV‐cherry injected mice (LV‐cherry: 28 d; LV‐PAK4: 35 d; *P* < .05) (Figure 9F).

### 3.8 Overexpression of PAK4 protects motor neurons from degeneration through enhancing CREB signalling in hSOD1G93A mice

**FIGURE 10:**
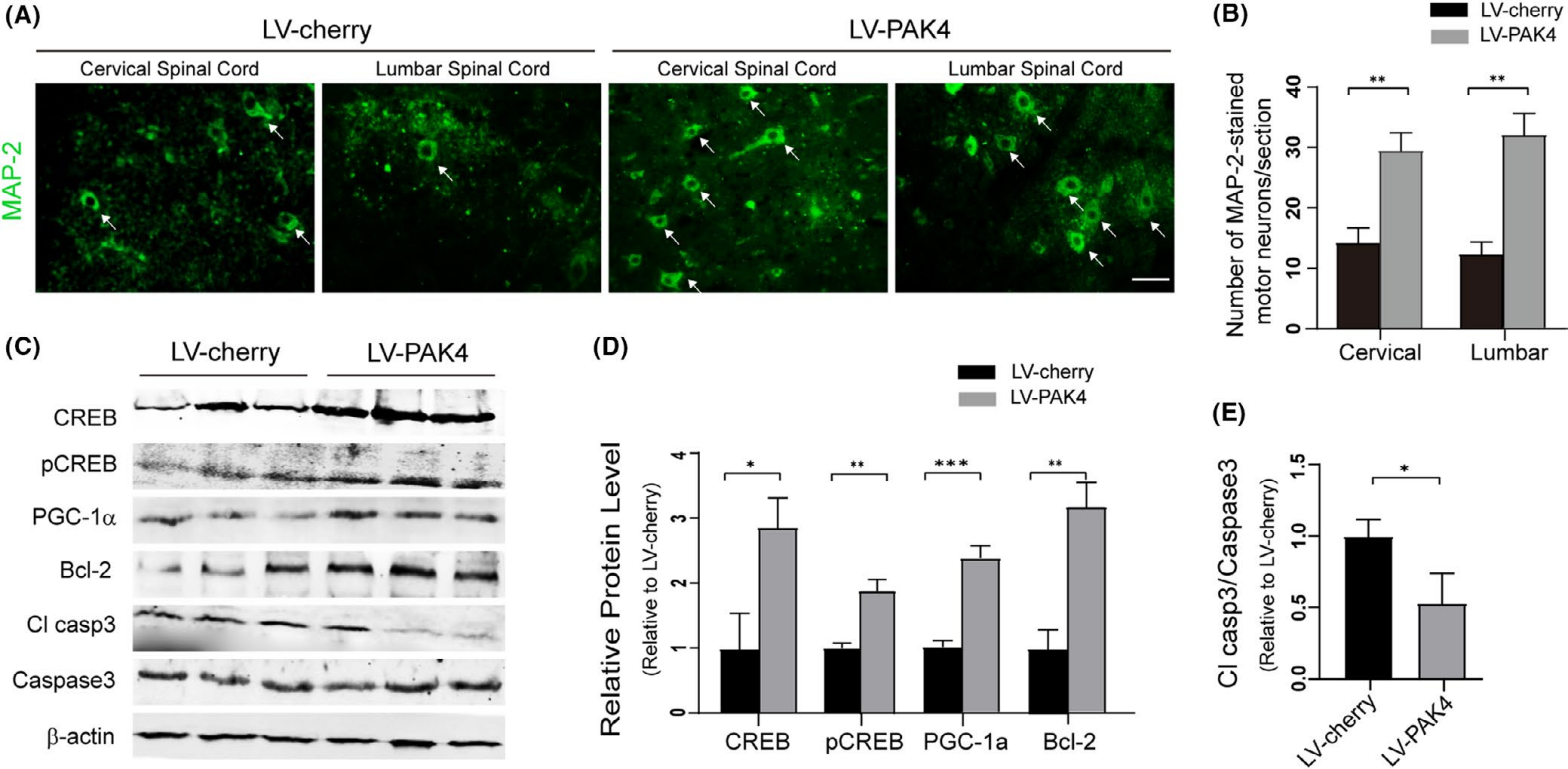
Overexpression of PAK4 in the spinal cord suppresses motor neuron degeneration by increasing the activity of the CREB signalling in hSOD1^G93A^ mice. A, Immunostaining of MAP‐2 in the VH of the spinal cords from LV‐cherry/LV‐PAK4 injected hSOD1^G93A^ mice at P135. B, Number of MAP‐2‐stained MN in the VH of LV‐cherry/LV‐PAK4 treated hSOD1^G93A^ mice. Scale bar = 50 μm. C, The levels of CREB, pCREB, PGC‐1a, Bcl‐2, caspase3 and cleaved‐caspase3 in the spinal cord from LV‐cherry/LV‐PAK4 injected hSOD1^G93A^ mice were analysed by Western blotting. D and E, Quantification analysis of protein levels of CREB, pCREB, PGC‐1a, Bcl‐2 and cleaved‐caspase3 normalized to β‐actin and caspase3. Data represent means ± SD, n = 3/group. Statistical analyses were performed using Student's *t*‐test. *P < .05, **P < .01, ***P < .001

To test the effects of PAK4 overexpression on MN degeneration, we performed immunostaining with anti‐MAP‐2 to examine the number of MN in the spinal cords of LV‐PAK4 treated hSOD1^G93A^ mice. We found that the number of MN surviving in LV‐PAK4‐injected hSOD1^G93A^ mice was significantly increased compared with LV‐cherry‐injected hSOD1^G93A^ mice (cervical spinal cord: 32.00 ± 3.61 vs 12.33 ± 2.08; lumbar spinal cord: 29.67 ± 3.06 vs 14.33 ± 2.52; *P* < .05; Figure 10A and B). Furthermore, immunoblotting analysis was performed to determine the effects of PAK4 overexpression on downstream consequences and neurodegeneration. Results revealed that LV‐PAK4 injected hSOD1^G93A^ mice exhibited high levels of CREB, pCREB, PGC‐1a and Bcl‐2 compared with LV‐cherry injected hSOD1^G93A^ mice (Figure 10C and D). Overexpression of PAK4 markedly decreased the levels of cleaved‐caspase3 in hSOD1^G93A^ mice (Figure 10C and E). These data suggested that PAK4 exerted neuroprotective effects by activating CREB signalling in the hSOD1^G93A^ mouse models of ALS.

## 4 DISCUSSION

Our results reveal that PAK4 is a potential effector of MN degeneration in ALS. We found that the expression and activation of PAK4 were downregulated in vivo and in vitro models of ALS. Overexpression of PAK4 protected MN from hSOD1^G93A^‐induced apoptosis. Notably, introducing LV‐PAK4 in hSOD1^G93A^ mice suppressed MN degeneration and thereby substantially extended mouse lifespan. Mechanistic studies revealed that CREB pathway in mSOD1 cells and hSOD1^G93A^ mice was inhibited and that the alteration was suppressed by overexpression of PAK4. We have also found that 3i, which inhibits CREB, compromised the ability of PAK4 to relieve mSOD1‐induced cytotoxicity. Taken together, our data suggested that PAK4 exerted neuroprotective effects for ALS MN by activating the CREB signalling.

PAK4 expresses ubiquitously and plays an essential role in multiple biological processes.^36^, ^59^ Dysregulation of PAK4 expression involves in various diseases.^60^ The functions of PAK4 in diseases have principally been investigated in cancers, and overexpression of it contributes to tumorigenesis.^61^, ^62^, ^63^ In the nervous system, PAK4 is required for neuronal development and axonal outgrowth.^35^, ^36^ In dopaminergic neurons of PD patients, the expression and activation of PAK4 are downregulated and this decline may promote PD pathogenesis.^24^ However, the relationship between PAK4 and ALS disease remains unclear. In this context, we demonstrate for the first time that PAK4 protein expression as well as activation significantly declined in cells and mice carrying hSOD1^G93A^. Considering that the expression of PAK4 declined at early‐sym stages, PAK4 might be involved in ALS disease progression.

A series of findings have shown that activated CREB promotes neuronal survival through a transcription‐dependent pathway.^14^, ^15^, ^16^, ^17^, ^20^ Moreover, the activation of CREB‐mediated downstream proteins plays a neuroprotective role in PD and AD rat models.^22^, ^24^, ^64^ Increasing expression of pCREB can improve MN function and prolong the life span of ALS mice.^28^ In accordance with previous studies, we found that PAK4 protected MN from mSOD1‐induced cytotoxicity through increasing CREB expression and activation. 3i ameliorated the neuroprotective effects of PAK4 and increased apoptosis by inhibiting the transcription activity and phosphorylation of CREB in ALS cell models. Thus, we further confirmed the protective role of CREB in the normal nervous system and neurodegenerative diseases.

Accumulating evidence suggests that CREB is a target of PAK4. In the present study, we found that knockdown of PAK4 reduced the levels and function of CREB in NSC34 cells. Overexpression of PAK4 increased CREB levels and function in mSOD1 cells. In mice and cell models of ALS, the low expression of PAK4 caused inhibition of the CREB pathway and promoted MN degeneration. Cumulatively, our findings are consistent with previous works that PAK4 sustains high CREB levels and improves CREB function in various tissues.^43^, ^44^ However, it is unclear how PAK4 maintains high levels of CREB. Additional studies are required to further elucidate the relationship between PAK4 and CREB expression and degradation. PAK4 could not directly phosphorylate CREB in vitro, suggesting that PAK4 activated CREB transcription by maintaining a high level. Whether PAK4 increases the transcriptional activity of CREB through a phosphorylation‐independent pathway in ALS needs subsequent study.

Growing evidence reveals that mitochondrial dysfunction plays a crucial role in ALS.^65^ PAK4 can inactivate the pro‐apoptotic protein BAD (members of the Bcl‐2 family pro‐apoptotic activities) by specifically phosphorylating S112.^66^ This phosphorylation protects cells from apoptosis by inhibiting BAD localization in the mitochondrial outer membrane. siRNA treatment decreased PAK4 expression in parallel with inhibition of the CREB downstream effector proteins PGC‐1a and Bcl‐2. Conversely, overexpression of PAK4 promoted the levels of Bcl‐2 and PGC‐1a in cell models of ALS. Moreover, 3i decreased PAK4‐induced expression of the CREB downstream proteins Bcl‐2 and PGC‐1a, thereby reducing cell survival. These data demonstrate that the up‐regulation of Bcl‐2 and PGC‐1a mediates PAK4‐induced neuroprotection. Considering the close association of PGC‐1a and Bcl‐2 with mitochondrial health,^67^, ^68^ PAK4 may contribute to mitochondrial protection in a transcription‐dependent manner. Thus, transcription‐dependent and transcription‐independent theories could explain the mechanisms that PAK4 protects mitochondria in the presence of neurotoxic stimuli.

Although PAK4 is a potential therapeutic target for ALS, persistent expression of it in the spinal cord after gene therapy might contribute to tumorigenesis because PAK4 is a potent oncogene. However, we did not monitor tumour formation in LV‐PAK4 injected hSOD1^G93A^ mice, but we did not maintain lengthy follow‐up. In addition, a lower cancer risk was observed in human ALS patients after diagnosis compared with healthy individuals.^69^ Thus, we speculate that a lack of tumorigenesis is likely due to a poor microenvironment for cell survival and proliferation in neurodegenerative diseases. ALS is characterized by progressive death of upper and lower MN, suggesting that the brain and spinal cord could not provide a proliferative environment. Moreover, most primary brain tumours develop from glial and meningeal cells. Thus, although PAK4 is regarded as an oncogenic gene, if a local delivery method can guarantee MN‐specific targeting, this could prevent undesirable effects and provide a promising therapeutic intervention for ALS. We also consider CREB as another target for modulation of the PAK4 neuroprotective pathway. CREB may be less potent than PAK4 because the role of CREB is more restricted compared with PAK4, but it may specifically enhance the expression of Bcl‐2 with a reduced risk of tumour formation.

Group I of the PAK proteins play a vital role in spinocerebellar ataxia type 1 (SCA1),^52^ fragile X syndrome (FXS),^70^ X‐linked mental retardation^71^ and neurodegenerative diseases such as HD^31^, ^32^ and AD.^72^ Rescue from disease pathology in cell and mice models of SCA1 and improvement of abnormal behaviour in FXS mice model by group I PAK inhibitors offer proof of PAKs as therapeutic targets.^73^, ^74^ Recent studies have implicated PAK4 and PAK6 in PD by demonstrating that PAK4 plays a neuroprotective role in PD models and that PAK6 is a downstream regulator of PD‐causing mutations.^24^, ^75^ Moreover, PAK signalling is stimulated by ALS2; mutations in the ALS2 gene cause some rare juvenile forms of ALS.^76^, ^77^ Our findings demonstrated that PAK4 plays a neuroprotective role in ALS models. In aggregate, these studies suggest that dysregulation of PAKs is a pathogenic mechanism in a series of neurological diseases. Thus, the development of more selective and effective interventions targeting PAKs may be a promising therapeutic strategy.

Many studies involving ALS are performed in one gender.^78^, ^79^, ^80^, ^81^ In this study, only female mice which exhibited less variability in lifetime were used.^82^, ^83^ Although the results of studies only including females or males are meaningful, disease pathways that only present in one gender may be potentially overshadowed by sex selection. In hSOD1^G93A^ mice, exercise was beneficial for females only.^83^ Male/female wild‐type mice show dramatic differences in response to a high‐fat diet,^84^ suggesting this diet may behave differently in ALS mice. Thus, in our further studies involving ALS, both males and females will be included, with sex being an additional variable analysed statistically. Other limitations are that we did not study how PAK4 expression levels correlate with ALS in human patients due to the absence of human postmortem brain samples. Further work needs to address the mechanism for PAK4 increasing the levels of CREB and determine if there are other mechanisms for PAK4 enhancing CREB transcriptional activity in ALS. Thus, the neuroprotective pathway of PAK4 still needs to be better clarified before targeting PAK4 as a therapeutic strategy in patients with ALS.

## 5 CONCLUSIONS

This work demonstrates that PAK4 protein levels and activation are downregulated in hSOD1^G93A^‐linked ALS cell and rat models, and overexpression of it protects MN from degeneration. The neuroprotective effect of PAK4 is mediated by activation of CREB‐mediated neuroprotective signalling pathway. miR‐9‐5p is responsible for the decreased expression of PAK4 in mSOD1cells. Our data show that PAK4 may be a potential therapeutic target in ALS.A

## AUTHOR CONTRIBUTIONS

Honglin Feng, Chaohua Cong, Weiwei Liang and Chunting Zhang conceived and designed the study. Chaohua Cong and Ying Wang conducted the experiments. Honglin Feng, Yueqing Yang, Xudong Wang and Shuyu Wang analysed the data and drafted the manuscript. Honglin Feng, Di Huo, Hongyong Wang and Di Wang participated in writing the manuscript. The authors read and approved the final manuscript.

## ETHICAL APPROVAL

All animal experiments were performed in accordance with the International Animal Care Guidelines and were approved by the Experimental Animal Ethics Committee of Harbin Medical University.

## Supporting information

Figure S1

Figure S2

Supplementary Material

## Data Availability

The datasets used during the current study are available from the corresponding author on reasonable request.

## References

[1] Zufiría M , Gil‐Bea FJ , Fernández‐Torrón R , et al. ALS: a bucket of genes, environment, metabolism and unknown ingredients. Prog Neurogibol. 2016;142:104‐129. 10.1016/j.pneurobio.2016.05.004 27236050

[2] Hardiman O , Al‐Chalabi A , Chio A , et al. Amyotrophic lateral sclerosis. Nat Rev Dis Primers. 2017;3:17085. 29052611 10.1038/nrdp.2017.85

[3] Clerc P , Lipnick S , Willett C . A look into the future of ALS research. Drug Discov Today. 2016;21(6):939‐949. 26861067 10.1016/j.drudis.2016.02.002

[4] Sreedharan J , Blair IP , Tripathi VB , et al. TDP‐43 mutations in familial and sporadic amyotrophic lateral sclerosis. Science. 2008;319(5870):1668‐1672. 18309045 10.1126/science.1154584 PMC7116650

[5] Byrne S , Walsh C , Lynch C , et al. Rate of familial amyotrophic lateral sclerosis: a systematic review and meta‐analysis. J Neurol Neurosurg Psychiatry. 2011;82(6):623‐627. 21047878 10.1136/jnnp.2010.224501

[6] Renton AE , Chiò A , Traynor BJ . State of play in amyotrophic lateral sclerosis genetics. Nat Neurosci. 2014;17(1):17‐23. 24369373 10.1038/nn.3584 PMC4544832

[7] Andersen PM , Al‐Chalabi A . Clinical genetics of amyotrophic lateral sclerosis: what do we really know?. Nat Rev Neurol. 2011;7(11):603‐615. 21989245 10.1038/nrneurol.2011.150

[8] Majoor‐Krakauer D , Willems PJ , Hofman A . Genetic epidemiology of amyotrophic lateral sclerosis. Clin Genet. 2003;63(2):83‐101. 12630951 10.1046/j.0009-9163.2002.00001.x

[9] Robberecht W , Philips T . The changing scene of amyotrophic lateral sclerosis. Nat Rev Neurosci. 2013;14(4):248‐264. 23463272 10.1038/nrn3430

[10] Gurney ME , Pu H , Chiu AY , et al. Motor neuron degeneration in mice that express a human Cu, Zn superoxide dismutase mutation. Science. 1994;264(5166):1772‐1775. 8209258 10.1126/science.8209258

[11] Rothstein JD . Current hypotheses for the underlying biology of amyotrophic lateral sclerosis. Ann Neurol. 2009;65(Suppl 1):S3‐S9. 19191304 10.1002/ana.21543

[12] Bensimon G , Lacomblez L , Meininger V . A controlled trial of riluzole in amyotrophic lateral sclerosis. ALS/Riluzole Study Group. N Engl J Med. 1994;330(9):585‐591. 8302340 10.1056/NEJM199403033300901

[13] Yoshino H , Kimura A . Investigation of the therapeutic effects of edaravone, a free radical scavenger, on amyotrophic lateral sclerosis (Phase II study). Amyotroph Lateral Scler. 2006;7(4):241‐245. 17127563 10.1080/17482960600881870

[14] Zhou Y , Won J , Karlsson MG , et al. CREB regulates excitability and the allocation of memory to subsets of neurons in the amygdala. Nat Neurosci. 2009;12(11):1438‐1443. 19783993 10.1038/nn.2405 PMC2783698

[15] Dong Y , Green T , Saal D , et al. CREB modulates excitability of nucleus accumbens neurons. Nat Neurosci. 2006;9(4):475‐477. 16520736 10.1038/nn1661

[16] Lopez de Armentia M , Jancic D , Olivares R , Alarcon JM , Kandel ER , Barco A . cAMP response element‐binding protein‐mediated gene expression increases the intrinsic excitability of CA1 pyramidal neurons. J Neurosci. 2007;27(50):13909‐13918. 18077703 10.1523/JNEUROSCI.3850-07.2007 PMC6673625

[17] Sargin D , Mercaldo V , Yiu AP , et al. CREB regulates spine density of lateral amygdala neurons: implications for memory allocation. Front Behav Neurosci. 2013;7:209. 24391565 10.3389/fnbeh.2013.00209 PMC3868910

[18] Lonze BE , Riccio A , Cohen S , Ginty DD . Apoptosis, axonal growth defects, and degeneration of peripheral neurons in mice lacking CREB. Neuron. 2002;34(3):371‐385. 11988169 10.1016/s0896-6273(02)00686-4

[19] Li S , Zhang C , Takemori H , Zhou Y , Xiong ZQ . TORC1 regulates activity‐dependent CREB‐target gene transcription and dendritic growth of developing cortical neurons. J Neurosci. 2009;29(8):2334‐2343. 19244510 10.1523/JNEUROSCI.2296-08.2009 PMC6666266

[20] Viosca J , Lopez de Armentia M , Jancic D , Barco A . Enhanced CREB‐dependent gene expression increases the excitability of neurons in the basal amygdala and primes the consolidation of contextual and cued fear memory. Learn Mem. 2009;16(3):193‐197. 19237641 10.1101/lm.1254209

[21] Chaturvedi RK , Hennessey T , Johri A , et al. Transducer of regulated CREB‐binding proteins (TORCs) transcription and function is impaired in Huntington's disease. Hum Mol Genet. 2012;21(15):3474‐3488. 22589249 10.1093/hmg/dds178 PMC3491961

[22] Jeong H , Cohen DE , Cui L , et al. Sirt1 mediates neuroprotection from mutant huntingtin by activation of the TORC1 and CREB transcriptional pathway. Nat Med. 2011;18(1):159‐165. 22179316 10.1038/nm.2559 PMC3509213

[23] Mabuchi T , Kitagawa K , Kuwabara K , et al. Phosphorylation of cAMP response element‐binding protein in hippocampal neurons as a protective response after exposure to glutamate in vitro and ischemia in vivo. J Neurosci. 2001;21(23):9204‐9213. 11717354 10.1523/JNEUROSCI.21-23-09204.2001 PMC6763920

[24] Won SY , Park MH , You ST , et al. Nigral dopaminergic PAK4 prevents neurodegeneration in rat models of Parkinson's disease. Sci Transl Med. 2016;8(367):367ra170. 10.1126/scitranslmed.aaf1629 27903866

[25] Yamashita M , Nonaka T , Hirai S , et al. Distinct pathways leading to TDP‐43‐induced cellular dysfunctions. Hum Mol Genet. 2014;23(16):4345‐4356. 24698978 10.1093/hmg/ddu152

[26] España J , Valero J , Miñano‐Molina AJ , et al. Beta‐Amyloid disrupts activity‐dependent gene transcription required for memory through the CREB coactivator CRTC1. J Neurosci. 2010;30(28):9402‐9410. 20631169 10.1523/JNEUROSCI.2154-10.2010 PMC6632424

[27] Herzog JJ , Xu W , Deshpande M , et al. TDP‐43 dysfunction restricts dendritic complexity by inhibiting CREB activation and altering gene expression. Proc Natl Acad Sci USA. 2020;117(21):11760‐11769. 32393629 10.1073/pnas.1917038117 PMC7260973

[28] Jeon GS , Im W , Shim YM , et al. Neuroprotective effect of human adipose stem cell‐derived extract in amyotrophic lateral sclerosis. Neurochem Res. 2016;41(4):913‐923. 26646002 10.1007/s11064-015-1774-z

[29] Yin X , Wang S , Wang X , et al. Lithium facilitates removal of misfolded proteins and attenuated faulty interaction between mutant SOD1 and p‐CREB (Ser133) through enhanced autophagy in mutant hSOD1G93A transfected neuronal cell lines. Mol Biol Rep. 2019;46(6):6299‐6309. 31529340 10.1007/s11033-019-05071-4

[30] Arias‐Romero LE , Chernoff J . A tale of two Paks. Biol Cell. 2008;100(2):97‐108. 18199048 10.1042/BC20070109

[31] Luo S , Mizuta H , Rubinsztein DC . p21‐activated kinase 1 promotes soluble mutant huntingtin self‐interaction and enhances toxicity. Hum Mol Genet. 2008;17(6):895‐905. 18065495 10.1093/hmg/ddm362

[32] Ma QL , Yang F , Frautschy SA , Cole GM . PAK in Alzheimer disease, Huntington disease and X‐linked mental retardation. Cell Logist. 2012;2(2):117‐125. 23162743 10.4161/cl.21602 PMC3490962

[33] Kreis P , Thévenot E , Rousseau V , Boda B , Muller D , Barnier JV . The p21‐activated kinase 3 implicated in mental retardation regulates spine morphogenesis through a Cdc42‐dependent pathway. J Biol Chem. 2007;282(29):21497‐21506. 17537723 10.1074/jbc.M703298200

[34] Nekrasova T , Jobes ML , Ting JH , Wagner GC , Minden A . Targeted disruption of the Pak5 and Pak6 genes in mice leads to deficits in learning and locomotion. Dev Biol. 2008;322(1):95‐108. 18675265 10.1016/j.ydbio.2008.07.006

[35] Dan C , Nath N , Liberto M , Minden A . PAK5, a new brain‐specific kinase, promotes neurite outgrowth in N1E–115 cells. Mol Cell Biol. 2002;22(2):567‐577. 11756552 10.1128/MCB.22.2.567-577.2002 PMC139731

[36] Qu J , Li X , Novitch BG , et al. PAK4 kinase is essential for embryonic viability and for proper neuronal development. Mol Cell Biol. 2003;23(20):7122‐7133. 14517283 10.1128/MCB.23.20.7122-7133.2003 PMC230313

[37] Mitra J , Hegde PM , Hegde ML . Loss of endosomal recycling factor RAB11 coupled with complex regulation of MAPK/ERK/AKT signaling in postmortem spinal cord specimens of sporadic amyotrophic lateral sclerosis patients. Mol Brain. 2019;12(1):55. 31196199 10.1186/s13041-019-0475-y PMC6567394

[38] Tan H , Chen M , Pang D , et al. LanCL1 promotes motor neuron survival and extends the lifespan of amyotrophic lateral sclerosis mice. Cell Death Differ. 2020;27(4):1369‐1382. 31570855 10.1038/s41418-019-0422-6 PMC7206132

[39] Kirby J , Ning K , Ferraiuolo L , et al. Phosphatase and tensin homologue/protein kinase B pathway linked to motor neuron survival in human superoxide dismutase 1‐related amyotrophic lateral sclerosis. Brain. 2011;134(Pt 2):506‐517. 21228060 10.1093/brain/awq345 PMC3030763

[40] Zhang C , Yang Y , Liang W , et al. Neuroprotection by urate on the mutant hSOD1‐related cellular and Drosophila models of amyotrophic lateral sclerosis: implication for GSH synthesis via activating Akt/GSK3β/Nrf2/GCLC pathways. Brain Res Bull. 2019;146:287‐301. 30690059 10.1016/j.brainresbull.2019.01.019

[41] Tyagi N , Bhardwaj A , Singh AP , McClellan S , Carter JE , Singh S . p‐21 activated kinase 4 promotes proliferation and survival of pancreatic cancer cells through AKT‐ and ERK‐dependent activation of NF‐κB pathway. Oncotarget. 2014;5(18):8778‐8789. 25238288 10.18632/oncotarget.2398 PMC4226721

[42] Kuijl C , Savage ND , Marsman M , et al. Intracellular bacterial growth is controlled by a kinase network around PKB/AKT1. Nature. 2007;450(7170):725‐730. 18046412 10.1038/nature06345

[43] Park MH , Lee HS , Lee CS , et al. p21‐Activated kinase 4 promotes prostate cancer progression through CREB. Oncogene. 2013;32(19):2475‐2482. 22710715 10.1038/onc.2012.255

[44] Yun CY , You ST , Kim JH , et al. p21‐activated kinase 4 critically regulates melanogenesis via activation of the CREB/MITF and β‐catenin/MITF pathways. J Invest Dermatol. 2015;135(5):1385‐1394. 25560280 10.1038/jid.2014.548

[45] Ryu BJ , Lee H , Kim SH , et al. PF‐3758309, p21‐activated kinase 4 inhibitor, suppresses migration and invasion of A549 human lung cancer cells via regulation of CREB, NF‐κB, and β‐catenin signalings. Mol Cell Biochem. 2014;389(1‐2):69‐77. 24366569 10.1007/s11010-013-1928-8

[46] Feng HL , Leng Y , Ma CH , Zhang J , Ren M , Chuang DM . Combined lithium and valproate treatment delays disease onset, reduces neurological deficits and prolongs survival in an amyotrophic lateral sclerosis mouse model. Neuroscience. 2008;155(3):567‐572. 18640245 10.1016/j.neuroscience.2008.06.040 PMC2709275

[47] Yin X , Ren M , Jiang H , et al. Downregulated AEG‐1 together with inhibited PI3K/Akt pathway is associated with reduced viability of motor neurons in an ALS model. Mol Cell Neurosci. 2015;68:303‐313. 26320681 10.1016/j.mcn.2015.08.009

[48] Yang YQ , Zheng YH , Zhang CT , et al. Wild‐type p53‐induced phosphatase 1 down‐regulation promotes apoptosis by activating the DNA damage‐response pathway in amyotrophic lateral sclerosis. Neurobiol Dis. 2020;134:104648. 31676238 10.1016/j.nbd.2019.104648

[49] Hunter DD , Cashman N , Morris‐Valero R , Bulock JW , Adams SP , Sanes JR . An LRE (leucine‐arginine‐glutamate)‐dependent mechanism for adhesion of neurons to S‐laminin. J Neurosci. 1991;11(12):3960‐3971. 1683902 10.1523/JNEUROSCI.11-12-03960.1991 PMC6575296

[50] Cashman NR , Durham HD , Blusztajn JK , et al. Neuroblastoma x spinal cord (NSC) hybrid cell lines resemble developing motor neurons. Dev Dyn. 1992;194(3):209‐221. 1467557 10.1002/aja.1001940306

[51] Durham HD , Dahrouge S , Cashman NR . Evaluation of the spinal cord neuron X neuroblastoma hybrid cell line NSC‐34 as a model for neurotoxicity testing. Neurotoxicology. 1993;14(4):387‐395. 7909362

[52] Park J , Al‐Ramahi I , Tan Q , et al. RAS‐MAPK‐MSK1 pathway modulates ataxin 1 protein levels and toxicity in SCA1. Nature. 2013;498(7454):325‐331. 23719381 10.1038/nature12204 PMC4020154

[53] Rupaimoole R , Calin GA , Lopez‐Berestein G , Sood AK . miRNA deregulation in cancer cells and the tumor microenvironment. Cancer Discov. 2016;6(3):235‐246. 26865249 10.1158/2159-8290.CD-15-0893 PMC4783232

[54] Xue J , Chen LZ , Li ZZ , Hu YY , Yan SP , Liu LY . MicroRNA‐433 inhibits cell proliferation in hepatocellular carcinoma by targeting p21 activated kinase (PAK4). Mol Cell Biochem. 2015;399(1–2):77‐86. 25410752 10.1007/s11010-014-2234-9

[55] Xia M , Wei J , Tong K . MiR‐224 promotes proliferation and migration of gastric cancer cells through targeting PAK4. Pharmazie. 2016;71(8):460‐464. 29442033 10.1691/ph.2016.6580

[56] Zeng B , Shi W , Tan G . MiR‐199a/b‐3p inhibits gastric cancer cell proliferation via down‐regulating PAK4/MEK/ERK signaling pathway. BMC Cancer. 2018;18(1):34. 29304764 10.1186/s12885-017-3949-2 PMC5756398

[57] Wang M , Gao Q , Chen Y , Li Z , Yue L , Cao Y . PAK4, a target of miR‐9‐5p, promotes cell proliferation and inhibits apoptosis in colorectal cancer. Cell Mol Biol Lett. 2019;24:58. 31728150 10.1186/s11658-019-0182-9 PMC6842216

[58] Waller R , Wyles M , Heath PR , et al. Small RNA sequencing of sporadic amyotrophic lateral sclerosis cerebrospinal fluid reveals differentially expressed miRNAs related to neural and glial activity. Front Neurosci. 2018;11:731. 29375285 10.3389/fnins.2017.00731 PMC5767269

[59] Abo A , Qu J , Cammarano MS , et al. PAK4, a novel effector for Cdc42Hs, is implicated in the reorganization of the actin cytoskeleton and in the formation of filopodia. EMBO J. 1998;17(22):6527‐6540. 9822598 10.1093/emboj/17.22.6527 PMC1171000

[60] Won SY , Park JJ , Shin EY , Kim EG . PAK4 signaling in health and disease: defining the PAK4‐CREB axis. Exp Mol Med. 2019;51(2):1‐9. 10.1038/s12276-018-0204-0 PMC6372590 30755582

[61] Radu M , Semenova G , Kosoff R , Chernoff J . PAK signalling during the development and progression of cancer. Nat Rev Cancer. 2014;14(1):13‐25. 24505617 10.1038/nrc3645 PMC4115244

[62] Fulciniti M , Martinez‐Lopez J , Senapedis W , et al. Functional role and therapeutic targeting of p21‐activated kinase 4 in multiple myeloma. Blood. 2017;129(16):2233‐2245. 28096095 10.1182/blood-2016-06-724831 PMC5399480

[63] Costa TDF , Zhuang T , Lorent J , et al. PAK4 suppresses RELB to prevent senescence‐like growth arrest in breast cancer. Nat Commun. 2019;10(1):3589. 31399573 10.1038/s41467-019-11510-4 PMC6689091

[64] Chang YM , Ashok Kumar K , Ju DT , et al. Dipeptide IF prevents the effects of hypertension‐induced Alzheimer's disease on long‐term memory in the cortex of spontaneously hypertensive rats. Environ Toxicol. 2020;35(5):570‐581. 31889399 10.1002/tox.22892

[65] Choi SY , Lopez‐Gonzalez R , Krishnan G , et al. C9ORF72‐ALS/FTD‐associated poly(GR) binds Atp5a1 and compromises mitochondrial function in vivo. Nat Neurosci. 2019;22(6):851‐862. 31086314 10.1038/s41593-019-0397-0 PMC6800116

[66] Gnesutta N , Qu J , Minden A . The serine/threonine kinase PAK4 prevents caspase activation and protects cells from apoptosis. J Biol Chem. 2001;276(17):14414‐14419. 11278822 10.1074/jbc.M011046200

[67] Shin JH , Ko HS , Kang H , et al. PARIS (ZNF746) repression of PGC‐1α contributes to neurodegeneration in Parkinson's disease. Cell. 2011;144(5):689‐702. 21376232 10.1016/j.cell.2011.02.010 PMC3063894

[68] Yuan J , Yankner BA . Apoptosis in the nervous system. Nature. 2000;407(6805):802‐809. 11048732 10.1038/35037739

[69] Fang F , Al‐Chalabi A , Ronnevi LO , et al. Amyotrophic lateral sclerosis and cancer: a register‐based study in Sweden. Amyotroph Lateral Scler Frontotemporal Degener. 2013;14(5‐6):362‐368. 23527497 10.3109/21678421.2013.775309 PMC5451142

[70] Hayashi ML , Rao BS , Seo JS , et al. Inhibition of p21‐activated kinase rescues symptoms of fragile X syndrome in mice. Proc Natl Acad Sci USA. 2007;104(27):11489‐11494. 17592139 10.1073/pnas.0705003104 PMC1899186

[71] Allen KM , Gleeson JG , Bagrodia S , et al. PAK3 mutation in nonsyndromic X‐linked mental retardation. Nat Genet. 1998;20(1):25‐30. 9731525 10.1038/1675

[72] Zhao L , Ma QL , Calon F , et al. Role of p21‐activated kinase pathway defects in the cognitive deficits of Alzheimer disease. Nat Neurosci. 2006;9(2):234‐242. 16415866 10.1038/nn1630

[73] Bondar VV , Adamski CJ , Onur TS , et al. PAK1 regulates ATXN1 levels providing an opportunity to modify its toxicity in spinocerebellar ataxia type 1. Hum Mol Genet. 2018;27(16):2863‐2873. 29860311 10.1093/hmg/ddy200 PMC6077814

[74] Dolan BM , Duron SG , Campbell DA , et al. Rescue of fragile X syndrome phenotypes in Fmr1 KO mice by the small‐molecule PAK inhibitor FRAX486. Proc Natl Acad Sci USA. 2013;110(14):5671‐5676. 23509247 10.1073/pnas.1219383110 PMC3619302

[75] Civiero L , Cirnaru MD , Beilina A , et al. Leucine‐rich repeat kinase 2 interacts with p21‐activated kinase 6 to control neurite complexity in mammalian brain. J Neurochem. 2015;135(6):1242‐1256. 26375402 10.1111/jnc.13369 PMC4715492

[76] Tudor EL , Perkinton MS , Schmidt A , et al. ALS2/Alsin regulates Rac‐PAK signaling and neurite outgrowth. J Biol Chem. 2005;280(41):34735‐34740. 16049005 10.1074/jbc.M506216200

[77] Hadano S , Hand CK , Osuga H , et al. A gene encoding a putative GTPase regulator is mutated in familial amyotrophic lateral sclerosis 2. Nat Genet. 2001;29(2):166‐173. 11586298 10.1038/ng1001-166

[78] Manjaly ZR , Scott KM , Abhinav K , et al. The sex ratio in amyotrophic lateral sclerosis: a population based study. Amyotroph Lateral Scler. 2010;11(5):439‐442. 20225930 10.3109/17482961003610853 PMC6485484

[79] Huisman MH , de Jong SW , van Doormaal PT , et al. Population based epidemiology of amyotrophic lateral sclerosis using capture‐recapture methodology. J Neurol Neurosurg Psychiatry. 2011;82(10):1165‐1170. 21622937 10.1136/jnnp.2011.244939

[80] Chiò A , Mora G , Calvo A , et al. Epidemiology of ALS in Italy: a 10‐year prospective population‐based study. Neurology. 2009;72(8):725‐731. 19237701 10.1212/01.wnl.0000343008.26874.d1

[81] Pape JA , Grose JH . The effects of diet and sex in amyotrophic lateral sclerosis. Rev Neurol (Paris). 2020;176(5):301‐315. 32147204 10.1016/j.neurol.2019.09.008 PMC8915943

[82] Shimojo Y , Kosaka K , Noda Y , Shimizu T , Shirasawa T . Effect of rosmarinic acid in motor dysfunction and life span in a mouse model of familial amyotrophic lateral sclerosis. J Neurosci Res. 2010;88(4):896‐904. 19798750 10.1002/jnr.22242

[83] Veldink JH , Bär PR , Joosten EA , Otten M , Wokke JH , van den Berg LH . Sexual differences in onset of disease and response to exercise in a transgenic model of ALS. Neuromuscul Disord. 2003;13(9):737‐743. 14561497 10.1016/s0960-8966(03)00104-4

[84] Pape JA , Newey CR , Burrell HR , et al. Per‐Arnt‐Sim Kinase (PASK) deficiency increases cellular respiration on a standard diet and decreases liver triglyceride accumulation on a western high‐fat high‐sugar diet. Nutrients. 2018;10(12):1990. 10.3390/nu10121990 PMC6316003 30558306

