# Supplementary figures and images for: PAK4 suppresses motor neuron degeneration in hSOD1G93A‐linked amyotrophic lateral sclerosis cell and rat models
Source: Cell Prolif. 2021 Feb 21;54(4):e13003. doi: 10.1111/cpr.13003 (PMC8016643; doi:10.1111/cpr.13003)

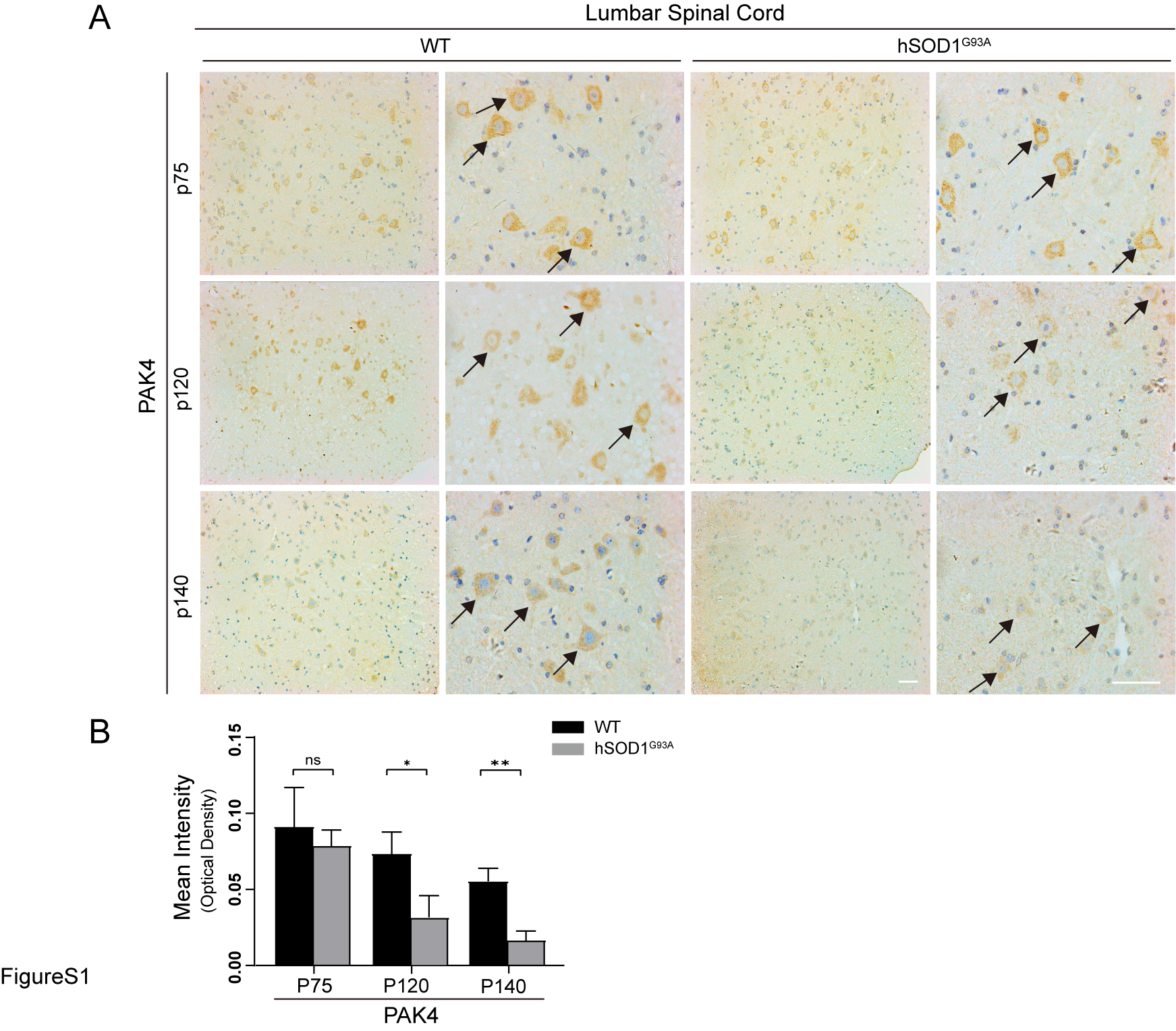

Supplement: Supplementary file 1 — Figure S1 [file CPR-54-e13003-s002.tif]

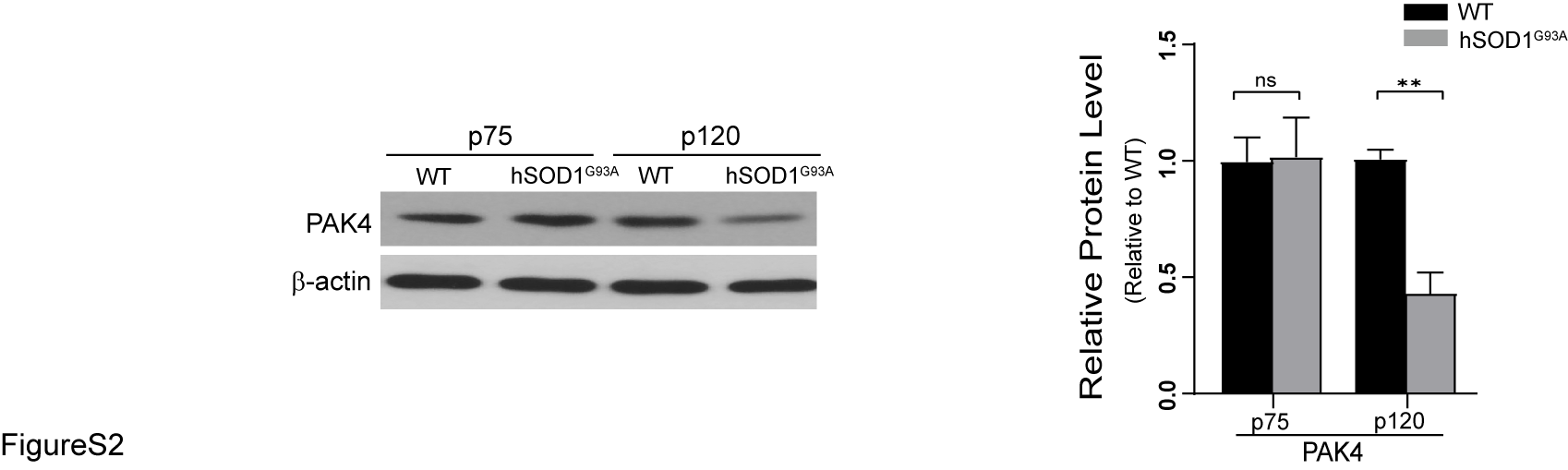

Supplement: Supplementary file 2 — Figure S2 [file CPR-54-e13003-s003.tif]
