# Supplementary material for: PAK4 suppresses motor neuron degeneration in hSOD1G93A‐linked amyotrophic lateral sclerosis cell and rat models
Source: Cell Prolif. 2021 Feb 21;54(4):e13003. doi: 10.1111/cpr.13003 (PMC8016643; doi:10.1111/cpr.13003)
Supplement: Supplementary file 3 — Supplementary Material [file CPR-54-e13003-s001.doc]

**Figure S1. The protein levels of PAK4 decreased in the lumbar spinal cords of hSOD1^G93A^ mice as the disease progressed.** Representative images (A) and OD values analysis (B) of PAK4-stained MN (indicated by black arrow) in the anterior horn of lumbar spianl cords at p75, p120 and p140. Scale bar = 50 μm, data were provided as means ± SD and were tested for significance using Student’s t test, ns ≥ 0.05, *p < 0.05, **p<0.01.

**Figure S2. The levels of PAK4 decreased in the spinal cords of hSOD1^G93A^ mice during disease progression.** Immunoblotting of spinal cord extract from p75 and p120 hSOD1^G93A^ mice and WT was conducted to detect PAK4 protein levels. Data were shown as means ± SD. Student’s t test was used to evaluate statistical significance, ns ≥ 0.05, *P < 0.05, **P < 0.01.
